# Key Role of the Ocean Western Boundary currents in shaping the Northern Hemisphere climate

**DOI:** 10.1038/s41598-019-39392-y

**Published:** 2019-02-28

**Authors:** Nour-Eddine Omrani, Fumiaki Ogawa, Hisashi Nakamura, Noel Keenlyside, Sandro W. Lubis, Katja Matthes

**Affiliations:** 1 Geophysical Institute, University of Bergen and Bjerknes centre for climate research, Bergen, Norway; 2 Research Center for Advanced Science and Technology, University of Tokyo, Tokyo, Japan; 3 Japan Agency for Marine-Earth Science and Technology, Yokohama, Japan; 4 Department of Geophysical Sciences, University of Chicago, Chicago, Illinois USA; 5 Research Division Ocean Circulation and Climate, GEOMAR Helmholtz Centre for Ocean Research, Kiel, Germany; 6 Kiel University, Kiel, Germany; 7 Nansen Environmental and Remote Sensing Center, Bjerknes Centre for Climate Research, Bergen, Norway

## Abstract

The individual impact of North Atlantic and Pacific Ocean Western Boundary Currents (OWBCs) on the tropospheric circulation has recently been studied in depth. However, their simultaneous role in shaping the hemisphere-scale wintertime troposphere/stratosphere-coupled circulation and its variability have not been considered. Through semi-idealized Atmospheric General-Circulation-Model experiments, we show that the North Atlantic and Pacific OWBCs jointly maintain and shape the wintertime hemispheric circulation and its leading mode of variability Northern Annular Mode (NAM). The OWBCs energize baroclinic waves that reinforce quasi-annular hemispheric structure in the tropospheric eddy-driven jetstreams and NAM variability. Without the OWBCs, the wintertime NAM variability is much weaker and its impact on the continental and maritime surface climate is largely insignificant. Atmospheric energy redistribution caused by the OWBCs acts to damp the near-surface atmospheric baroclinicity and compensates the associated oceanic meridional energy transport. Furthermore, the OWBCs substantially weaken the wintertime stratospheric polar vortex by enhancing the upward planetary wave propagation, and thereby affecting both stratospheric and tropospheric NAM-annularity. Whereas the overall impact of the extra-tropical OWBCs on the stratosphere results mainly from the Pacific, the impact on the troposphere results from both the Pacific and Atlantic OWBCs.

## Introduction

The physical understanding of the extra-tropical atmospheric impact on the Ocean Western Boundary Currents (OWBCs) has greatly advanced since the classic pioneering works^1–3^. In contrast, the understanding of the impact of Northern Hemisphere (NH) OWBCs on the atmospheric circulation has advanced only recently and has focused mainly on the individual impact of the Gulf Stream and Kuroshio/Oyashio Currents on the tropospheric circulation and on small, local and basin scales^4–9^. Compared to the Northern hemisphere, a recent idealized aqua-planet model simulations indicate that Southern Hemisphere mid-latitude SST fronts impact the hemispheric-scale atmospheric circulation and its dominant variability^10–14^. For the Northern Hemisphere there is emerging evidence that the Gulf Stream in the Atlantic and Kuroshio/Oyashio Currents in the Pacific locally maintain and anchor storm-tracks, precipitation and basin-scale tropospheric circulation^4,8,15^. However, the combined role of the North Atlantic and Pacific OWBCs and their individual contributions in maintaining the hemispheric-scale circulation and NAM variability in stratospheric/troposphere-coupled system remains unclear.

It has been argued that the dynamical coupling with the ocean is not an essential feature of the intrinsic dynamics of North Atlantic Oscillation (NAO) or NAM^16,17^. The NAM and NAO exert a big impact on the surface continental climate^18,19^, ocean circulation^20,21^, and the marine and terrestrial ecosystems^22,23^. It has been established that the NAM and NAO are essentially an internal mode of atmospheric variability governed primarily by interactions between midlatitude westerlies and eddies (synoptic-scale cyclones and anticyclones), as it can be simulated in AGCMs with climatological SSTs prescribed at the lower boundary^24^. The coupling to the ocean is therefore not considered essential for the NAM (NAO)^16,17,25^. Nonetheless, the coupling to the ocean and hence ocean circulation is very important and largely influences the SST-distributions used in those AGCM-studies. In fact, recent idealized AGCM-studies show that organization of those eddies into storm-tracks is influenced by the SST-fronts along the OWBCs^10–12^. This influence is understandable from linear wave-theory^26,27^, which suggests that cyclones and anticyclones recurrently develop where the sharp gradient of near-surface air-temperature is maintained^28^. This temperature gradient is directly linked to the SST fronts through turbulent heat fluxes at the ocean surface^29^.

The aim here is (i) to identify the joint impact of the north Atlantic and Pacific OWBCs on the hemispheric-scale circulation in both troposphere and stratosphere using semi-idealized model experiments and (ii) to clarify the implications for the Northern Hemisphere climate, including energy budget and climate variability. We focus on the features of large-scale circulation that can be largely simulated with stratospheric-resolving IPCC-class models: i.e., the mid-latitude tropospheric eddy-driven jets, stratospheric polar vortex, and associated dynamics and variability. By considering the climatological SST and continents, this work can be seen as an extension of the previous highly idealized aqua-planet studies and the studies with idealized continents^10–14^.

Unlike its Southern Hemisphere counterpart, the impact of Northern Hemisphere OWBCs on the atmosphere tends to be masked by the corresponding impacts of land-sea thermal contrasts and large–scale orography^8,30^; thus it is difficult to isolate the OWBC-impacts through conventional AGCM-experiments or observational analysis. We therefore utilize several sets of semi-idealized AGCM-experiments similar to^10–12^, but under more realistic Northern Hemisphere conditions using the Hamburg stratosphere resolving AGCM MAECHAM5^31^ (Method).

## Results

### Impact on the planetary-scale troposphere-stratosphere coupled circulation

**Figure 1:**
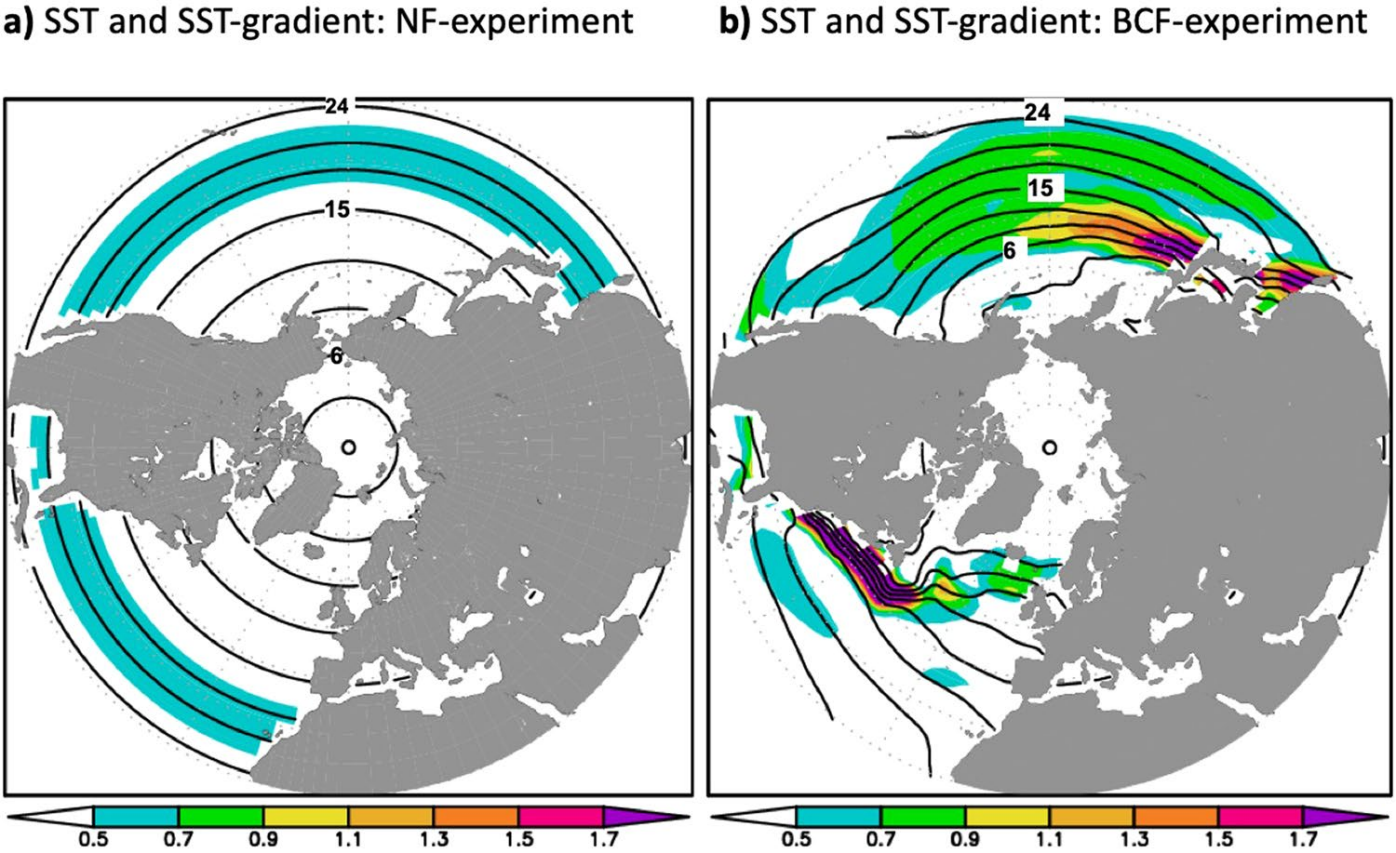
SST-forcing: January SST and its meridional gradients are plotted for (**a**) non-front (NF)-experiment and (**b**) both (Atlantic and Pacific) climatological SST fronts (BCF) experiment (Method).

In order to assess the combined impact of the North Pacific and Atlantic OWBCs, we conducted a set of two experiments. The first experiment (BCF-experiment, Method) is conducted using observed climatological SSTs with observed extra-tropical SST-fronts^32^ only in the North Atlantic and Pacific (Fig. 1b). In order to isolate the capability of the Northern Hemisphere OWBCs in reproducing the planetary-scale circulation in the BCF-experiment from other important factors,

we removed the tropical SST-asymmetry, the impact of the sea-ice and the mid-latitude SST-front in the Southern Hemisphere (Method, SFig. 1c). In the second experiment (NF-experiment), the mid-latitude SST-fronts over the Atlantic and Pacific basins are also removed (Fig. 1a, Method). The Northern extra-tropical SST-fronts, which represent the regions of highest meridional SST-gradient, form in general as a confluence of cool OWBCs of subpolar gyres (East Greenland/Labrador Currents in the Atlantic and the Oyashio Current in the Pacific that transport cool water southward) with warm OWBCs of subtropical gyres (Gulf Stream in the Atlantic and Kuroshio Current in the Pacific that transport warm tropical water northward). The removal of the SST-fronts can thus be interpreted as reduction of the thermal confluence that results in cooling (warming) along the OWBCs of subtropical (subpolar) gyres (SFig. 1b).

The BCF-experiment is able to reproduce the key features of the large-scale atmospheric circulation seen in the reanalysis, including storm-tracks, tropospheric eddy driven jets, the stratospheric polar night jet and the propagation of synoptic waves patterns (SFigs 2–4). There are however some discrepancies with respect to observations, for example the low-level jet maximum is shifted eastward and somewhat stronger than in the NCEP-reanalysis (SFig. 3). These are partly related to the semi-idealized surface boundary conditions. For example, the consideration of the tropical SST-asymmetry (Methode, SFigs 2–4) in an additional experiment (BCF_Tro-Experiment), can shift the position of Pacific surface jet and its magnitude more towards the surface jet in the NCEP-reanalysis. Most of the common large-scale atmospheric features shared by the BCF_Trop-Experiment and NCEP-reanalysis are maintained mainly by the Northern Hemisphere SST-front (SFigs 2–4). The intermediate resolution used reproduces thus the mean futures of the large-scale atmospheric circulation with discrepancies that are not large compared to those found in other climate models having moderate resolution^33^. The remaining discrepancies form the NCEP-reanalysis in our experiments can be related to the missing sea-ice, model error, Southern Hemisphere SST-fronts and intermediate resolution used. Thus, we are confident that our experimental design can be useful in understanding key features of winter-time atmospheric circulation simulated by current IPCC-class climate models.

**Figure 2:**
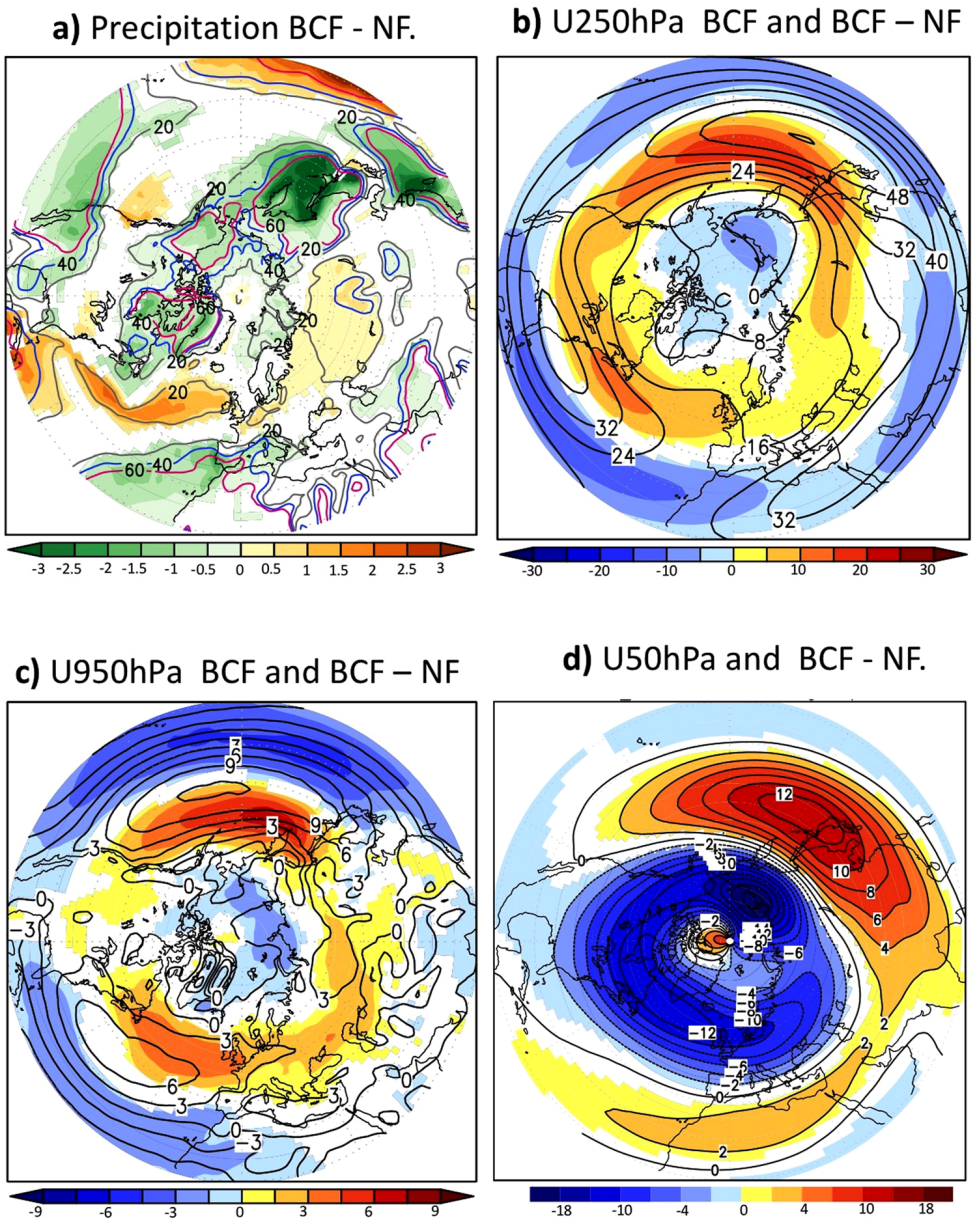
Atmospheric response to the OWBCs: Shading in (**a**–**d**) represents the wintertime (JFM)-response to SST fronts (i.e., BCF minus NF experiments) for precipitation (mm/day), zonal wind (m/s) at 250 hPa, 950 hPa and 50 hPa, respectively. The contours in (**b**,**c**) show the corresponding BCF-experiment climatology. In (**a**), the grey, blue and red lines enclose the areas where the local response of BCF-experiment to SST fronts exceeds 20%, 30% and 60% of the BCF-experiment climatological values, respectively. Only significant differences at 95%-level are shaded according to a two-tailed t-test.

The comparison of the BCF- and NF-experiment reveal that the North Atlantic and Pacific OWBCs exert a noticeable impact on the distribution of precipitation (Fig. 2a). Along the Gulf Stream, a well-organized precipitation band is simulated in agreement with^4^. This may contribute to maintaining the storm-track^20^ and thereby generating planetary waves through latent heat release^34^. The impacts of the OWBCs are not confined to the vicinities of the Gulf Stream and Kuroshio/Oyashio Currents but extend to the surrounding continents. The associated precipitation change reaches more than 60% of the BCF-experiment climatology over several Northern Hemisphere regions (Fig. 2a in contours).

The combined impact of the North Atlantic and Pacific OWBCs on the wintertime Northern Hemisphere tropospheric circulation is recognized as a planetary-scale strengthening of the westerlies in mid-to-high latitudes and a weakening in lower latitudes (Fig. 2b,c). This response corresponds to poleward shift of the Atlantic and Pacific tropospheric eddy-driven jet streams, with a projection onto the positive NAM in the Northern Hemisphere and specifically the NAO in the Atlantic sector. The presence of the Atlantic and Pacific OWBCs has thus an impact on the quasi-annular structure of the tropospheric eddy-driven jets. The associated response can account for more than 90% (60%) of the climatological westerlies in the BCF-experiment at the lower (upper) tropospheric levels (SFig. 5). The North Atlantic and Pacific OWBCs jointly act to weaken the planetary-scale wintertime stratospheric polar vortex and surrounding westerlies and thus warm the polar stratosphere (Fig. 2d and SFig. 6).

### The dynamics of the joint and individual impacts of the Atlantic and Pacific OWBCs

**Figure 3:**
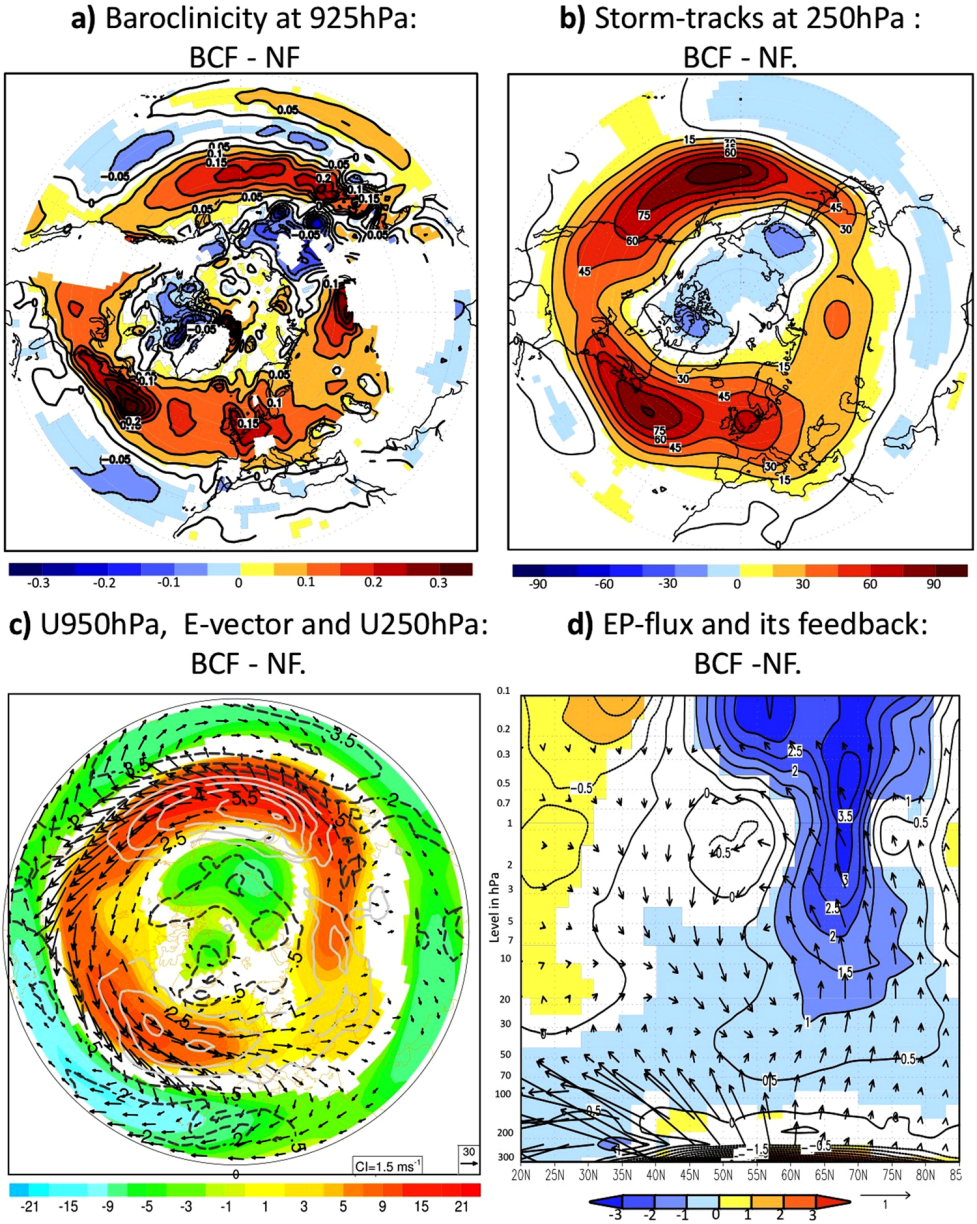
Dynamics of the Northern Hemisphere atmospheric response to the OWBCs: (**a**–**c**) represent the wintertime response to SST-front of: (**a**) the baroclinicity (in 1/day), (**b**) storm-tracks and (**c**) horizontal components of E-vector (vectors, see Method) superposed on the tropospheric zonal wind at 250 hPa (shaded in m/s) and 950 hPa (white contour, in m/s). (**d**) represents the response of the stratospheric EP-flux (vector, see Method) and its feedback on the zonally averaged zonal wind (shaded in (m/s)/day). In all panels, only significant differences at 95%-level according to a two-tailed t-test are shaded.

**Figure 4:**
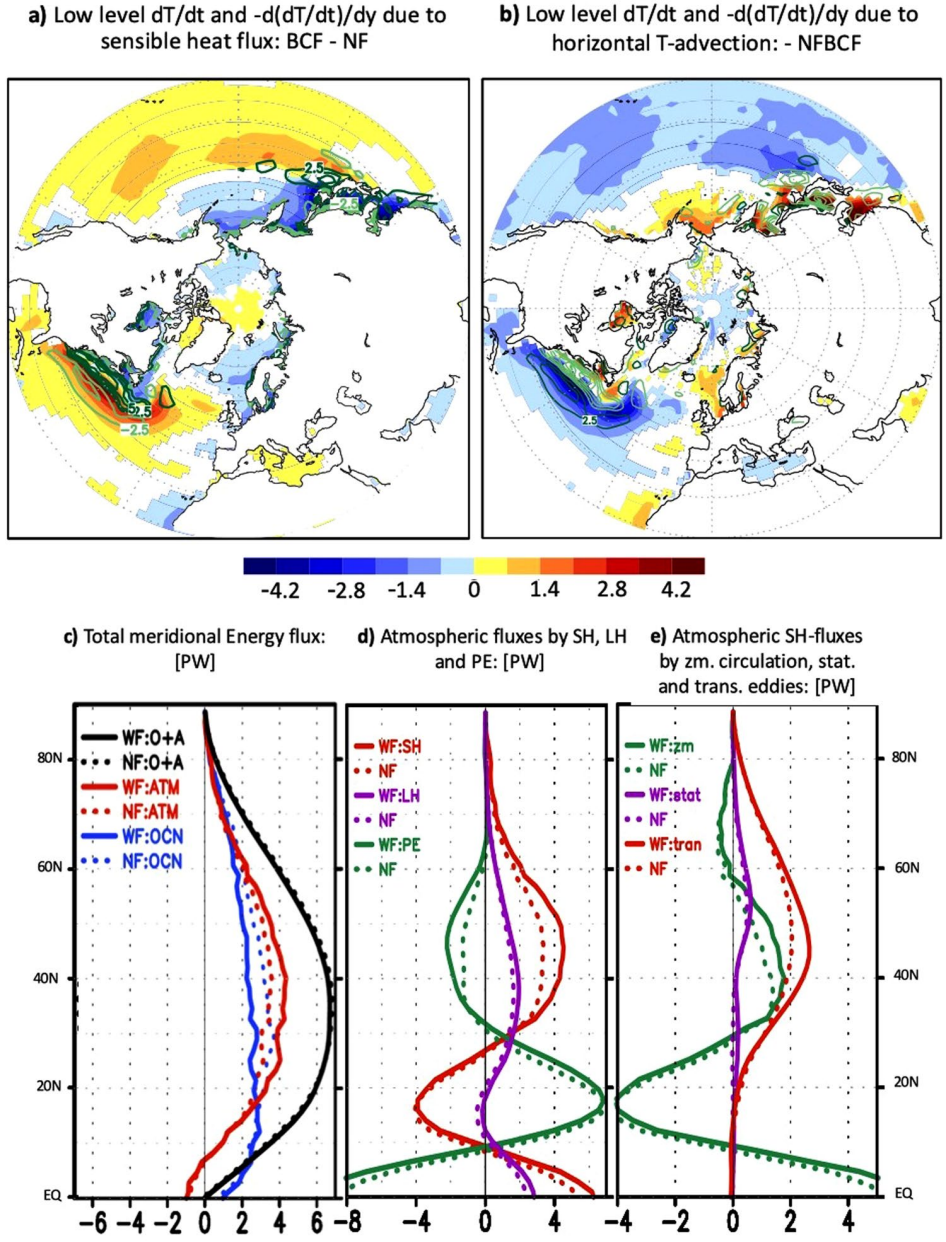
Energetic perspective of the Northern Hemisphere atmospheric response to OWBCs: (**a**) represents the wintertime response to the SST fronts (BCF- NF) of the lower-tropospheric temperature tendency (shaded, in K/day, see Method) due to the upward surface sensible heat flux superposed on its reversed (i.e. equatorward) gradient (contour in (K/day)/°latitude), and (**b**) represents the lower-tropospheric temperature tendency (shaded, in K/day, see Methods) due to horizontal thermal advection superimposed on its equatorward gradient (green contour in (K/day)/°latitude). The contours representing the equatorward temperature gradients in (**a**,**b**) are illustrated in dark green for positive values and light green for negative values. (**c**) represents the overall climatological mean of the zonally-averaged poleward total energy transport (in PW) for the BCF-experiment (solid lines) and NF-Experiment (dashed lines). The total poleward energy transport in (**a**) is computed for the atmosphere/ocean-coupled system (black), only by the atmosphere (red) and only by the ocean (blue, see Method). (**d**) Represents the decomposition of the total atmospheric poleward energy transport into sensible heat (SH, red), latent heat (LH, blue) and potential energy (green). The transport of the atmospheric kinetic energy is much smaller compared to the other terms and therefore neglected. (**e**) represents the decomposition of the atmospheric poleward heat transport into the contributions from the transient eddies (red), stationary eddies (blue) and steady mean meridional circulation (green). Only significant differences at 95%-level according to two-tailed t-tests are shaded for the tendencies in (**a**,**b**).

Synoptic-scale baroclinic disturbances play a crucial role in the extra-tropical tropospheric general circulation. These cyclones and anticyclones mediate the impacts of the OWBCs on the atmospheric circulation (Fig. 3a-c), consistent with linear wave-theory^26,27^ and previous studies^5,6,10–12^. The sharp SST gradients, associated with the inter-gyre heat confluence, maintain strong lower-tropospheric temperature gradients through their control of sensible and latent heat release from the ocean (Fig. 4a and SFig. 7a). Consequently, the baroclinicity^28^, (Method, Fig. 3a), which is proportional (inversely proportional) to the meridional temperature gradient or vertical wind shear (static stability), is also enhanced. The effective contribution of latent heat release to the near-surface baroclinicity has been shown to be weak^29^, since condensational heating occurs mostly in the mid-to-upper troposphere in association with cloud-formation. The latent heating can be important for the development of individual storms^28^. The storm-tracks (defined as regions of large variance of high-pass filtered geopotential height) are thus enhanced along the SST-fronts (Fig. 3b), showing local intensification of poleward eddy heat flux, as a measure of the baroclinic eddy development (Method, SFig. 7b).

The enhanced storm-track activity can in turn explain the response of the tropospheric westerlies to the SST-fronts, as diagnosed with the baroclinic wave activity flux called E-vector (Method)^28,35^. The E-vector is used to diagnose the feedback forcing by baroclinic waves on the background zonal flow. Strong divergence of the E-vector in mid-to-high latitudes and convergence in lower latitudes indicate an enhanced eddy-induced acceleration and deceleration of the westerlies in response to the OWBCs (Fig. 3c), respectively. The associated equatorward E-vector corresponds to increased westerly momentum transfer from the subtropical jet into the eddy-driven polar-front-jet (Method), consistently with the stronger (weaker) mid-to-high (lower) latitude westerlies. In the stratosphere, the weakened polar vortex is maintained by the enhanced injection of planetary wave-activity from the troposphere and its convergence in the mid-to-high-latitude stratosphere (Fig. 3d)

The overall tropospheric adjustment acts to offset the OWBC-maintained baroclinicity (due to turbulent heat fluxes), in which the horizontal atmospheric circulation advects warmer air into the cooler areas and vice versa (Fig. 4a,b). The transient (mainly synoptic) eddies are thereby the primary contributor to the relaxation of the meridional temperature gradient through their poleward heat transport (SFig. 8a–c), which dominates the total meridional temperature advection. The adjustment is also effective indirectly through the zonal temperature advection by the wave-induced mean zonal wind-response (SFig. 8d,e). This effect is characteristic of the Northern Hemisphere SST fronts and cannot be realized in the aqua-planet AGCM-experiments^10–12^, which mimic the Southern Hemisphere situation.

In the zonally averaged framework, the adjustment of the atmosphere to the OWBCs follows the concept of the Bjerknes compensation (Fig. 4c–e, Method). Each SST pattern reflect in general the pattern of the oceanic heat and energy transport that maintains it. Such energy transport can be computed from the energy balance of the Earth-atmosphere system (Method). Removing the sharp SST-gradients is associated with an increase in the oceanic poleward energy transport (Fig. 4c) that is consistent with the warmer high latitude and cooler low-to-mid latitude SSTs, and hence with reduction of the SST gradient along the confluence zone of the OWBCs (SFig. 1b). The enhanced oceanic energy transport is compensated by a reduction of the atmospheric energy transport under the Bjerknes compensation^36^, which is accounted for by the reduced poleward transport of sensible heat by transient eddies (Fig. 4d,e). As discussed above, the adjustments of the transient eddies to the SST fronts are manifested largely as the response of synoptic-scale eddies (SFig. 8a,b).

**Figure 5:**
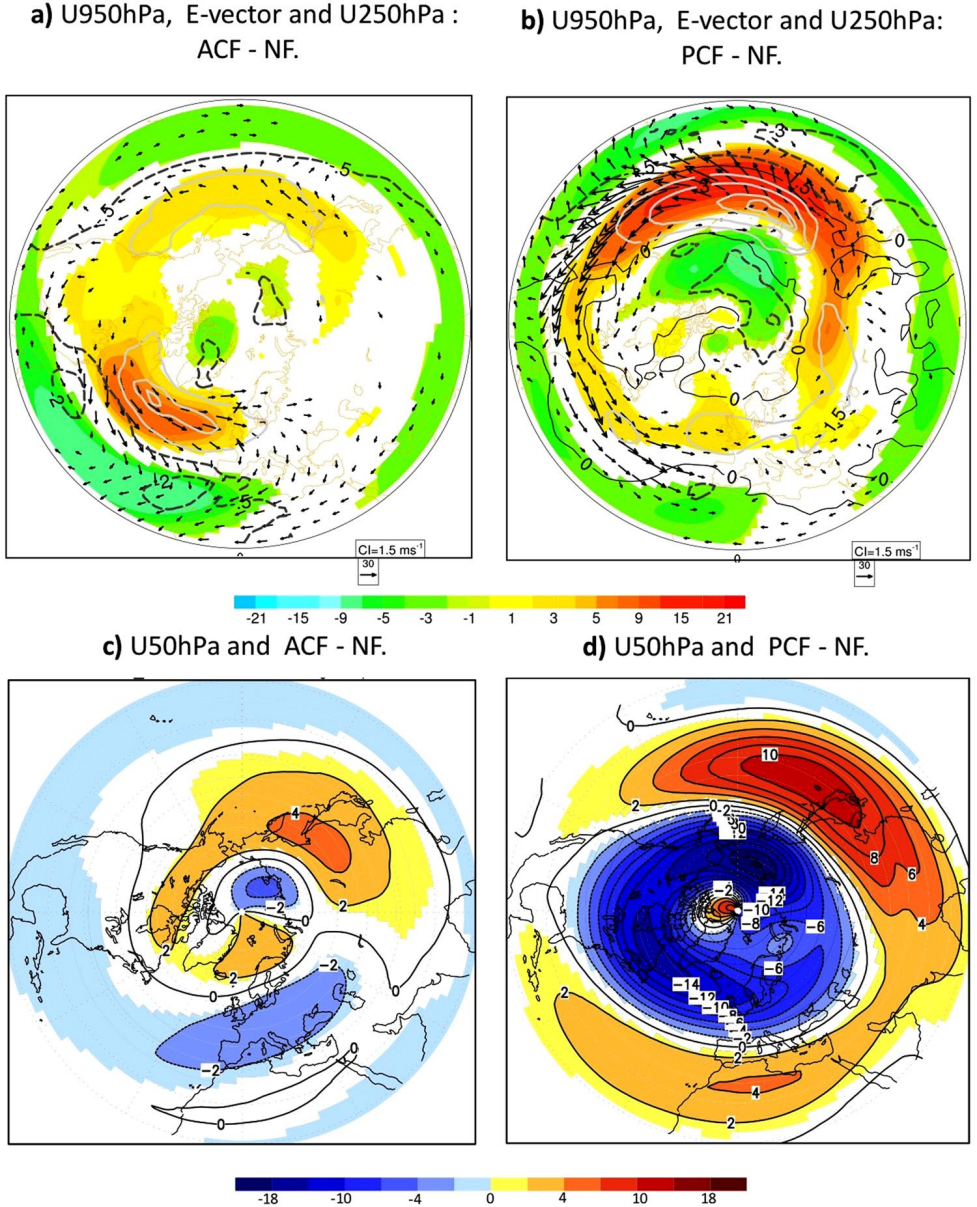
The atmospheric response to North Atlantic and Pacific OWBCs individually: (**a**,**b**) are similar to Fig. 3c but for the contribution from (**a**) the Atlantic (ACF-NF) and (**b**) Pacific (PCF-NF) SST fronts to the overall tropospheric response (BCF-NF) in Fig. 3c. (**c**,**d**) are identical to Fig. 2d but for the contributions of the Atlantic (ACF-NF) and Pacific (PCF-NF) to the overall (BCF-NF) stratospheric response in Fig. 2d.

To isolate the individual contributions from the Atlantic and Pacific OWBCs, we performed two additional experiments with the climatological SST-fronts only in the Atlantic (ACF-experiment) and only in the Pacific (PCF-experiment, Method). Regarding the contributions from the individual ocean basins, the overall strengthening of the tropospheric eddy-driven polar-front jets results from both Pacific and Atlantic OWBCs (Fig. 5a,b, SFig. 9a–f), whereas the overall weakening of the stratospheric polar night-jet results mainly from the Pacific OWBC (Fig. 5c,d, SFig. 9g–i). The strengthening of the tropospheric westerlies due to the Pacific OWBCs is not confined to the Pacific but extends into the Atlantic as well as the American and Eurasian continents, in the form of circumpolar teleconnection (Fig. 5b). Although somewhat weaker, a similar inter-basin circumpolar teleconnection is also seen as a response to the Atlantic OWBCs (Fig. 5a).

### Relevance of the OWBCs for the NAM

**Figure 6:**
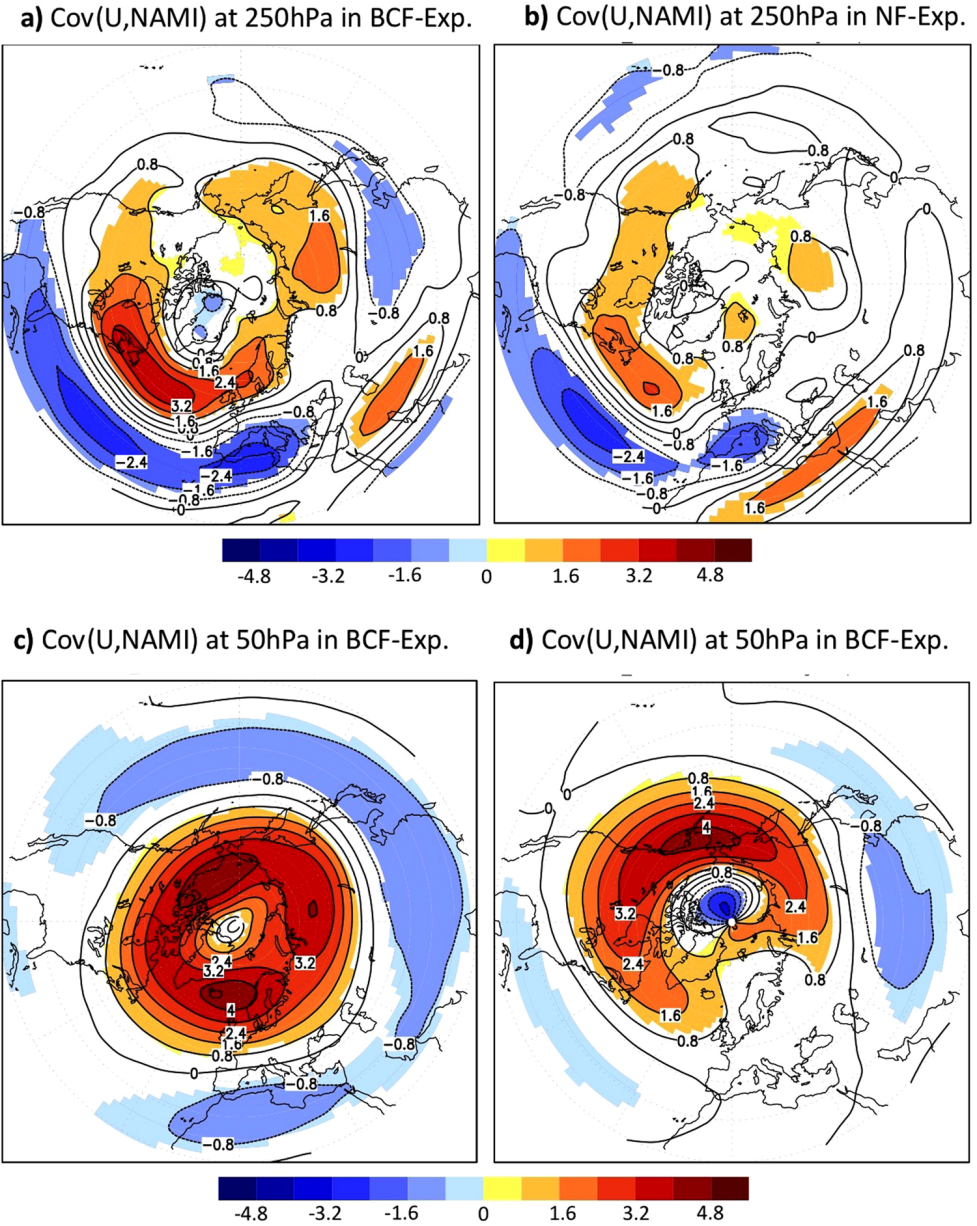
Implications for Northern Annular Mode: (**a**,**b**) represent the covariance of the wintertime 250 hPa westerly wind with the wintertime 250 hPa NAMI for (**a**) BCF-experiment and (**b**) NF-experiment. (**c**,**d**) are the same as (**a**,**b**) but for the covariance of 50 hPa westerly wind with the 250 hPa NAMI. Only significant differences at 95%-level according to a covariance test are shaded.

**Figure 7:**
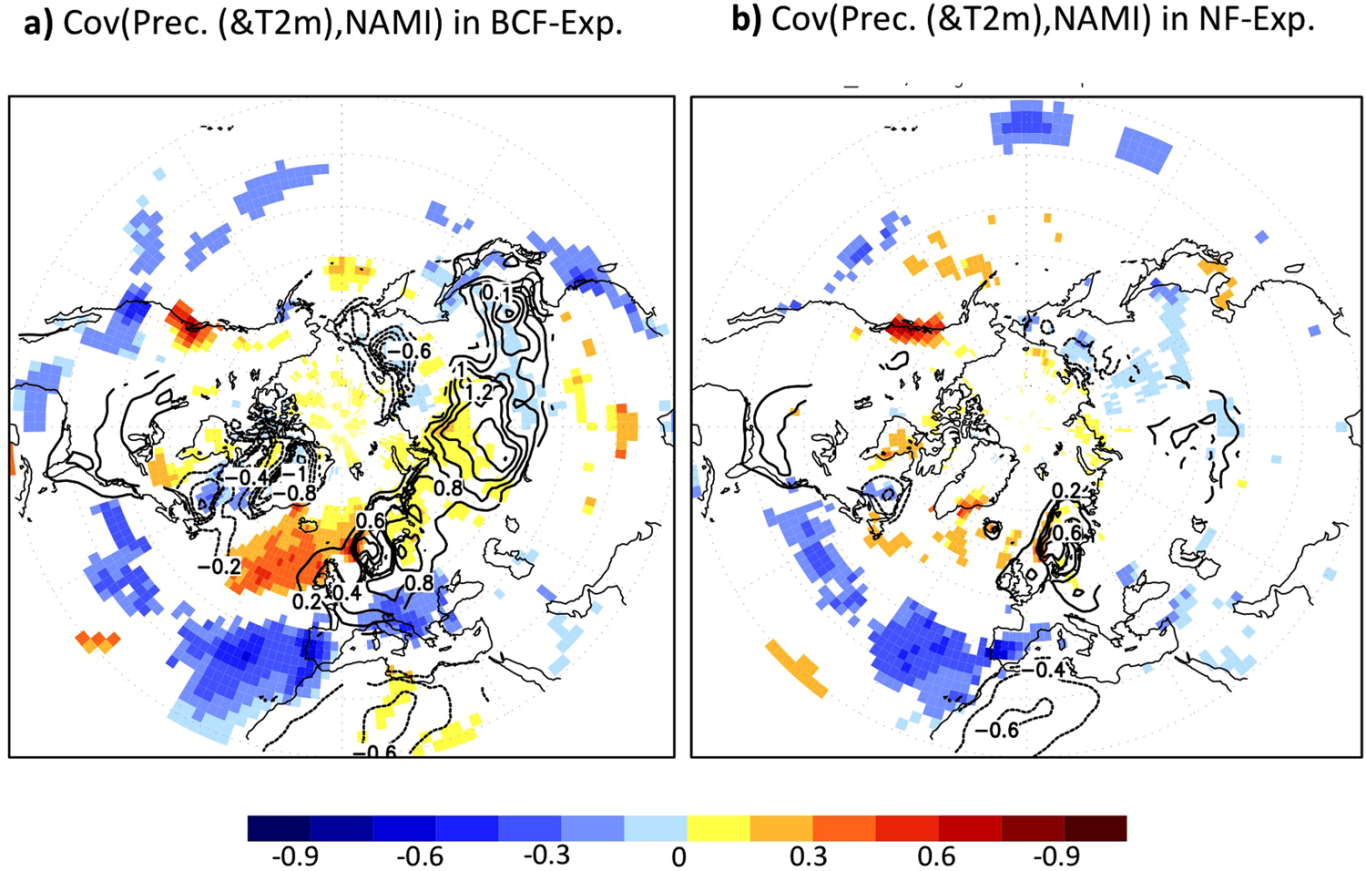
Implications for Northern Hemispher climate: (**a**,**b**) represent the covariance of wintertime two-meter temperature (contour, in K) and precipitation (shading, in mm/day) with the wintertime 250 hPa NAMI. Only significant differences at the 95%-level according to a covariance test are shaded for the surface temperature and contoured for the precipitation.

The impact of the OWBCs on the Hemisphere-scale atmospheric circulation can result in significant impact of the Ocean on the intrinsic dynamics and the structure of the NAM or NAO. Giving the smaller simulated NAM variability compared to the reanalysis, the observed overall structures of tropospheric and stratospheric NAM^18^ can be reproduced simply by the Northern Hemisphere climatological SST-front prescribed in the presence of continents (Fig. 6 and SFig. 11). In the absence of the OWBCs and the associated climatological SST-fronts, the NAM (or NAO) influence on Eurasia would become insignificant over large regions (Figs 6 and 7). The NAM-induced fluctuations in tropospheric zonal winds and their eastward extension into the mid-to-high latitude Eurasia are reduced substantially (as much as 50%) in the NF-experiment as compared to the BCF-experiment (Fig. 6a,b). The OWBCs also act to enhance the annular structure of the NAM in both stratosphere and troposphere (Fig. 6a–d). The differences in the NAM-structure are perceptible in surface climate (Fig. 7a,b). In the absence of the sharp SST-gradients, the NAM-induced fluctuations in precipitation and surface temperature would become insignificant over most of the mid-to-high latitude regions including Eurasia, Eastern and Western Atlantic, and Mediterranean areas. The OWBCs can thus considerably enhance the annularity of the NAM and its importance for surface climate variability.

## Concluding Remarks

The recent years were marked by considerable progress in understanding the impact of the OWBCs on the atmospheric circulation^4–9^. However, some fundamental questions have not been addressed. In particular, it is still unknown how the Atlantic and Pacific OWBCs control simultaneously the planetary-scale stratosphere/troposphere-coupled mean circulation, the NAM and the resulting Northern Hemisphere surface climate variability in observations and standard IPCC model simulations with moderate horizontal resolution. Our semi-idealized experiments show that the OWBCs associated with the Gulf Stream and the Kuroshio/Oyashio Currents are important in shaping both the stratospheric and the tropospheric hemisphere-scale circulation and its leading mode of variability. In the troposphere, both Atlantic and Pacific OWBCs maintain the strong westerly Northern Hemisphere eddy-driven polar-front-jets through turbulent heat release with a response projecting on the NAM. This heat release restores and thus maintains the near-surface baroclinicity efficiently against the eddy-induced atmospheric heat transport. This restoration, in turn, is necessary for recurrent storm development and thereby the formation of storm-tracks and the maintenance of the strong westerly eddy-driven polar-front-jets over both the Atlantic and Pacific basins. Both local response and circumpolar inter-basin teleconnection are important for the quasi-annular structure of the westerly eddy-driven jet response to the Atlantic and Pacific OWBCs. In the stratosphere, a weaker and warmer polar vortex is attributable mainly to enhanced upward planetary-wave propagation in the presence of the Pacific OWBCs. Regarding the atmospheric variability, the North Pacific and Atlantic OWBCs act to enhance the annular structure of the NAM in the stratosphere/troposphere-coupled system and significantly increase the NAM-impact on surface climate. The NAM- or NAO-like tropospheric response and the maintenance of the stratosphere/troposphere-coupled circulation by OWBCs can also be simulated using zonally symmetric SST-fronts analogous to^10–12^ (SFig. 1, Method, SFig. 10). Such significant NAM- or NAO-like response can in turn feedback on the ocean, through changes in wind-driven Northern Hemisphere oceanic gyres^1–3^ and Atlantic thermohaline circulation^20^. In this way, the Northern Hemisphere OWBCs can be seen as an important interface that links the large-scale atmospheric and oceanic circulations and thus contributes in shaping the mean state and variability of the coupled atmosphere/ocean-system. The simulation of realistic SSTs along the OWBCs and reduction of the still existing SST-bias along the NH-OWBCs in climate models^37^ may thus be important for an adequate climate-prediction and climate-change projection.

Our results can help to understand the first-order dynamical impact of the OWBCs on the planetary-scale circulation and its variability that can be captured by IPCC-class models with standard moderate spatial resolutions. The potential impact of the fine structures in the SST-fronts and ocean eddies requires AGCMs with higher resolution and can thus not be inferred from our study.

## Methods

### Experimental design

In this study, we performed a set of experiments using the stratosphere-resolving Hamburg version of the European Centre Atmospheric General Circulation Model (AGCM): MAECHAM5^31^. We used the horizontal resolution of T63 (equivalent to ~180 km grid intervals) and 39 vertical levels going up to 0.01 hPa. The lower-boundary condition of the AGCM was taken from the observed climatology of monthly-mean SSTs from 1950 through 2008 (HadISST)^32^. Here we investigate the impact of the Northern Hemisphere OWBCs on the atmospheric circulation that can be simulated with model resolutions currently used to study climate (i.e., as in the IPCC AR6). The OWBCs maintain local maxima in the SST-gradients that we call SST-fronts (Fig. 1b and SFig. 1c). The OWBCs maintain the SST-fronts through the heat confluence associated with boundary currents of both subtropical and subpolar oceanic gyres. We isolate the impact of the OWBCs by performing AGCM experiments with prescribed SST gradient modified in different ways. Firstly, we produced a Non-Front (NF) SST profile by taking the zonal average of the observed SSTs and smoothing its mid-latitude frontal gradient in both hemispheres. In the NF-Experiment, the observed zonally averaged SST-gradient was relaxed to a constant value northward and southward of the tropical flank of the SST fronts (33.5N and 33.5S). Thereby, a linear poleward SST decrease was assumed, in such a way that the polar SST reaches the freezing temperature (Fig. 1a; black line in SFig. 1c). The effect of sea ice is removed by removing the Arctic and Antarctic sea-ice. This method of smoothing the SST-fronts is similar to the method used in^11–14^ for aqua-planet configurations.

In order to isolate the impact of the Northern Hemisphere OWBCs on the large-scale atmospheric circulation, we further performed six sensitivity experiments with realistic climatological SST (ACF, PCF and BCF) and more idealized zonally symmetric (or uniform) SST fronts (ASF, PSF and BSF).

The first experiment (BCF-experiment) is conducted using observed climatological SSTs with realistic climatological extra-tropical SST fronts in both, the Atlantic and the Pacific (Fig. 1b); otherwise the experiment is identical to the NF-experiment. The BCF-experiment is compared against the NF-experiment (Fig. 1a and SFig. 1c) to extract the joint impacts of the Atlantic and Pacific OWBCs on the atmosphere. To identify the individual contributions from the Atlantic and Pacific OWBCs, two additional experiments are compared against the NF-experiment. The ACF-experiment isolates the impact of the Atlantic OWBCs using the observed climatological SST-front only in the Atlantic and smoothed SST conditions in the Pacific; while the converse PCF-experiment isolates the impact of the Pacific OWBCs. The SSTs are identical to the NF-experiments outsides these prescribed SST-fronts regions. Similar simulations were conducted using zonally symmetric (or zonally uniform) instead of climatological SST fronts. In the Atlantic zonally-uniform SST-front experiment (ASF-experiment), the previously described climatological Atlantic SST front is replaced by averaging the observed climatological SST longitudinally across the western Atlantic between 78°W and 32°W; and the SST in the PSF-experiment is similarly obtained by zonally averaging the climatological SST across the Pacific (140°E and 170°E). BSF refers to Both symmetric Fronts with the Atlantic and Pacific zonally-uniform SST fronts are both prescribed. Since the tropical SST also impacts the Northern Hemisphere circulation significantly, we also performed an additional experiment with realistic tropical SST asymmetry and Northern Hemisphere SST-fronts (BCF_Tro-Experiment). The BCF_Tro-Experiment is used in addition to BCF_experiment to test the capability of our model to reproduce the mean large-scale features discussed in this study. The comparison of this experiment with the SST-fronts experiments and reanalysis will give more insight about the role of OWBCs in maintaining the planetary-scale circulation.

In all SST front experiments a meridional linear interpolation in the equatorial flank of the imposed frontal area was performed in order to avoid discontinuity in the SST profile between the front and non-front regions. Similar linear interpolations in the polar region were performed but in the zonal direction in ASF, PSF, ACF and PCF experiments, in order to avoid the inter-basin discontinuity. Because of the similarity of the atmospheric response in the realistic and symmetric SST-front experiments (SFig. 10), only the more realistic climatological SST-front experiments will be considered in our main discussions.

### Data Analysis

#### Mean state and variability

All AGCM experiments were integrated for at least 50 years after 3-year spin-up, and the response is defined as the difference in the January-February-March mean between a given front-experiment and the NF experiment. A two-tailed t-test was performed to assess statistical significance of the response. In order to understand the impact of the OWBCs on the variability of the coupled stratosphere/troposphere-system, the Northern Annular Mode (NAM) is extracted as the leading Empirical Orthogonal Function (EOF) mode of geopotential height north of 20N^18^. The NAM-index (NAMI), defined as the corresponding principal component time series, was regressed locally onto various meteorological quantities, in order to assess the impact of the OWBCs on NAM-associated climate variability.

#### Atmospheric dynamics

Baroclinic eddies: The baroclinicity is measured locally as a quantity^28^ that is proportional to the meridional air temperature gradient and inversely proportional to the static stability. The baroclinic wave-activity (or storm-track activity) is diagnosed with the 3-dimensional wave-activity flux called E-vector^28^. The storm-track activity is measured as the variance of high-pass filtered geopotential height fluctuations (in periods of 1–12 day), and the E-vector with ${E}_{x}=\overline{{v^{\prime} }^{2}-{u^{\prime} }^{2}}$, ${E}_{y}=\overline{-u^{\prime} v^{\prime} }$ *and* ${E}_{z}=\frac{{f}_{0}\overline{\theta ^{\prime} v^{\prime} }}{-(\partial {\rm{\Theta }}/\partial p)}$^35^ can be used to diagnose the feedback forcing by baroclinic waves on the background zonal flow. In the definition, *u* (*v*) is the zonal (meridional) velocity and *θ* (*Θ*(*p*)) potential temperature (its standard, i.e., hemispherically-averaged, vertical distribution) and the bar denotes time averaging. Only high-pass-filtered fluctuations (with periods shorter than ~12 day) are used for evaluating the E-vector. Its convergence (divergence) indicates eddy-induced acceleration (deceleration) of the westerlies, and its equatorward component $(i.e.,{E}_{y}=-\,\overline{u^{\prime} v^{\prime} } < 0)$is indicative of the eddy momentum flux from the subtropics into midlatitude eddy-driven jet.

#### Forcing of the near-surface baroclinicity

In this work, the forcing of the baroclinicity in the planetary boundary layer (PBL) by sensible heat flux (SHF) is computed analogously to^12^ as equatorward gradient of the temperature tendency between 1000 and 850 hPa due to the upward heat flux: $\frac{\partial (-g/\,({C}_{p}{\rm{\Delta }}p)\times SHF))}{\partial y}$, where Δ*p* = 150 *hpa* and *C*_*p*_ is the atmospheric specific heat at constant pressure. In order to understand the atmospheric adjustment due to the OWBCs, the lower-tropospheric (1000-850 hPa) horizontal temperature- (*T*-) advection $(-u\frac{\partial T}{\partial x}-v\frac{\partial T}{\partial y})$ was computed and decomposed (Fig. 4b and SFig. 8) into individual contributions from the transient eddies ($-u^{\prime} \frac{\partial T^{\prime} }{\partial x}-v^{\prime} \frac{\partial T^{\prime} }{\partial y}$, inclusive baroclinic eddies) and time-mean circulation $(-\bar{u}\frac{\partial \bar{T}}{\partial x}-\bar{v}\frac{\partial \bar{T}}{\partial y})$. We also decomposed the horizontal *T*-advection into contributions from the zonal $(-u\frac{\partial T}{\partial x})$ and meridional $(-v\frac{\partial T}{\partial y})$advection. The effect of the horizontal *T*-advection on the low-level baroclinicity was measured as the 1000-850 hPa mean equatorward gradient of the *T*-tendency due to the *T*-advection.

Bjerknes compensation and poleward energy flux: The Bjerknes compensation states: if the energy flux at the top of the atmosphere and the oceanic heat storage are quasi-stationary, the total energy transport of the climate system should not vary much. This means that the energy transport of the atmosphere and the ocean should compensate each other^36^. In order to understand the role of the oceanic fronts in modulating the poleward energy flux of the climate system, we computed the zonally averaged meridional energy flux of the atmosphere (*F*_*A*_) and the ocean (*F*_*O*_) based on the energy balance of the Earth-atmosphere system^38,39^. Here we assume a steady state, where the net radiative flux at the top of the atmosphere (*R*_*top*_) is balanced by the convergence of the meridional heat transport by both the atmosphere and the ocean $(-\frac{\partial ({F}_{A}+{F}_{O})}{\partial y})$^38,39^. The meridional energy flux in the atmosphere is computed directly from the atmospheric variables as the sum of potential, latent, sensible and kinetic energy transport. The equivalent oceanic meridional energy transport (expected from the climatological SST-configuration used) is obtained as residual^38,39^. The contribution of the kinetic energy is negligible compared to the other forms of energy and will not be presented. The meridional sensible heat flux is decomposed into the contributions from steady mean meridional circulation, transient and stationary eddies^38,39^.

Stratospheric dynamics: The stratospheric changes through planetary waves can be diagnosed through the Eliassen-Palm (EP) flux in its primitive form (page 128 Eq. 3.5.3(a) and (b) in^40^). In the quasi-geostrophic approximation, the vertical component of the EP-flux is proportional to the poleward eddy heat transport and its meridional component is proportional to the equatorward eddy zonal momentum transport. Convergence of the EP-flux in the high-latitude stratosphere means weakening of the westerly polar-night jet during NH winter and warming in the stratospheric polar vortex.

## Supplementary information

Key Role of the Ocean Western Boundary currents in shaping the Northern Hemisphere climate.

## Data Availability

The datasets generated during and/or analysed during the current study are available from the corresponding author on reasonable request.
