# Author Correction: Key Role of the Ocean Western Boundary currents in shaping the Northern Hemisphere climate

**DOI:** 10.1038/s41598-020-60548-8

**Published:** 2020-02-20

**Authors:** Nour-Eddine Omrani, Fumiaki Ogawa, Hisashi Nakamura, Noel Keenlyside, Sandro W. Lubis, Katja Matthes

**Affiliations:** 1 Geophysical Institute, University of Bergen and Bjerknes centre for climate research, Bergen, Norway; 2 Research Center for Advanced Science and Technology, University of Tokyo, Tokyo, Japan; 3 Japan Agency for Marine-Earth Science and Technology, Yokohama, Japan; 4 Department of Geophysical Sciences, University of Chicago, Chicago, Illinois USA; 5 Research Division Ocean Circulation and Climate, GEOMAR Helmholtz Centre for Ocean Research, Kiel, Germany; 6 Kiel University, Kiel, Germany; 7 Nansen Environmental and Remote Sensing Center, Bjerknes Centre for Climate Research, Bergen, Norway

Correction to: *Scientific Reports* 10.1038/s41598-019-39392-y, published online 28 February 2019

This Article contains errors. In Figure 4b, ‘NFBCF’ should read ‘BCF-NF’.

In addition, in Figure 6d, ‘BCF-Exp’ should read ‘NF-Exp’. Furthermore, in the legend of Figure 6,

“(**c**,**d**) are the same as (**a**,**b**) but for the covariance of 50 hPa westerly wind with the 250 hPa NAMI.”

should read:

“(**c**,**d**) are the same as (**a**,**b**) but for the covariance of 50 hPa westerly wind with the 50 hPa NAMI.”

The correct Figures 4 and 6 appear below as Figures 1 and 2.

**Figure 1:**
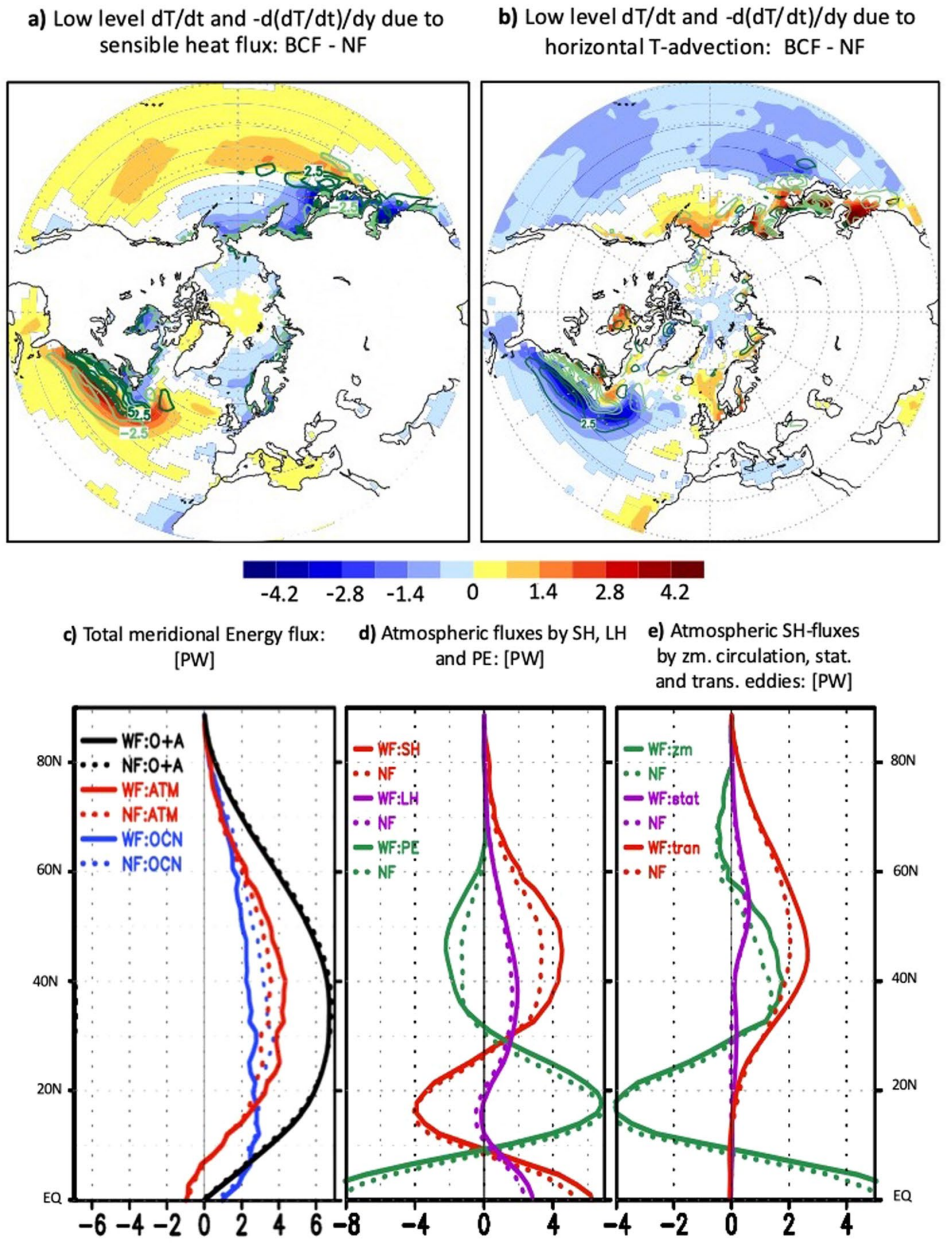
Energetic perspective of the Northern Hemisphere atmospheric response to OWBCs: (**a**) represents the wintertime response to the SST fronts (BCF- NF) of the lower-tropospheric temperature tendency (shaded, in K/day, see Method) due to the upward surface sensible heat flux superposed on its reversed (i.e. equatorward) gradient (contour in (K/day)/°latitude), and (**b**) represents the lower-tropospheric temperature tendency (shaded, in K/day, see Methods) due to horizontal thermal advection superimposed on its equatorward gradient (green contour in (K/day)/°latitude). The contours representing the equatorward temperature gradients in (**a,b**) are illustrated in dark green for positive values and light green for negative values. (**c**) represents the overall climatological mean of the zonally-averaged poleward total energy transport (in PW) for the BCF-experiment (solid lines) and NF-Experiment (dashed lines). The total poleward energy transport in (**a**) is computed for the atmosphere/ocean-coupled system (black), only by the atmosphere (red) and only by the ocean (blue, see Method). (**d**) Represents the decomposition of the total atmospheric poleward energy transport into sensible heat (SH, red), latent heat (LH, blue) and potential energy (green). The transport of the atmospheric kinetic energy is much smaller compared to the other terms and therefore neglected. (**e**) represents the decomposition of the atmospheric poleward heat transport into the contributions from the transient eddies (red), stationary eddies (blue) and steady mean meridional circulation (green). Only significant differences at 95%-level according to two-tailed t-tests are shaded for the tendencies in (**a,b**).

**Figure 2:**
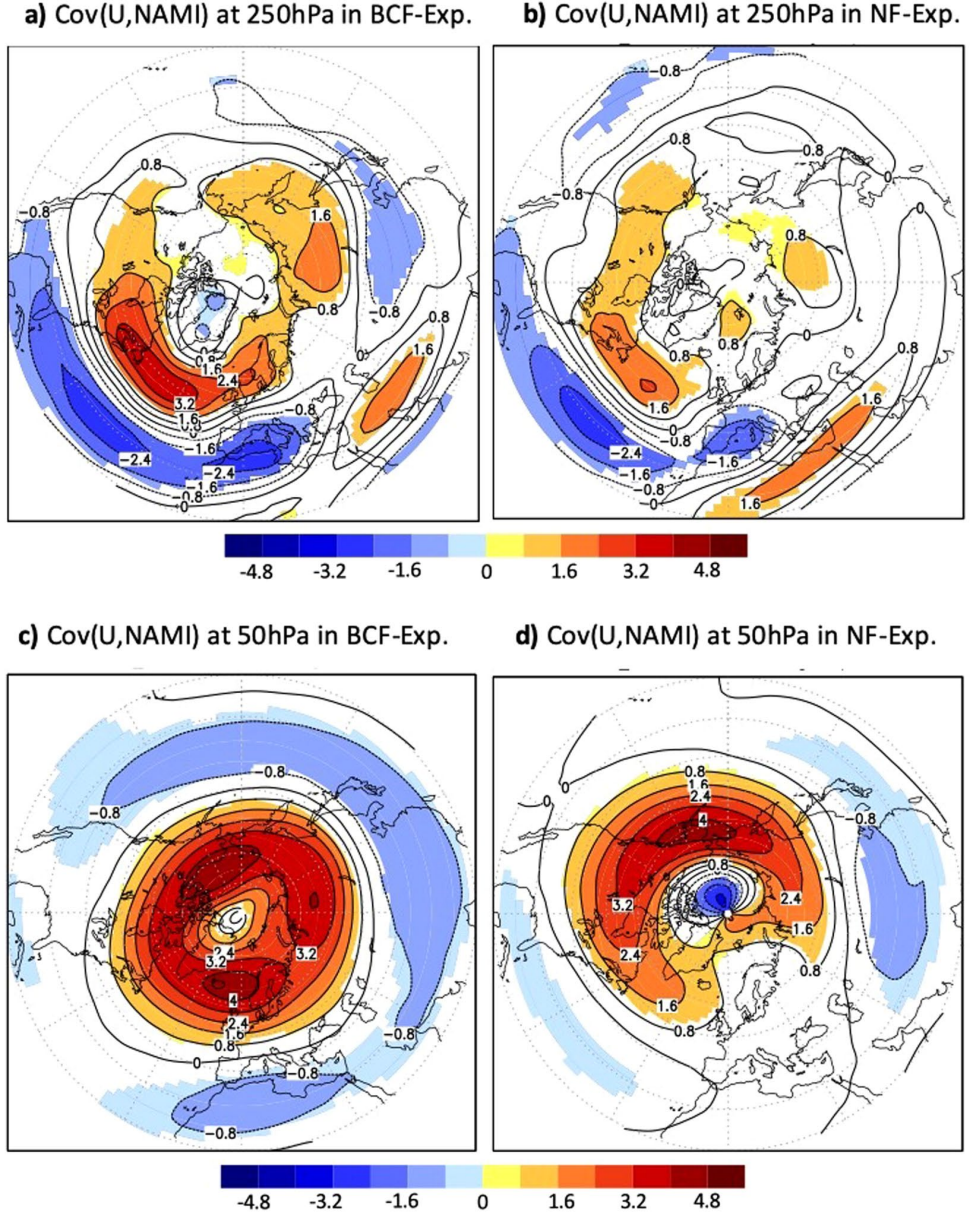
Implications for Northern Annular Mode: (**a,b**) represent the covariance of the wintertime 250 hPa westerly wind with the wintertime 250 hPa NAMI for (**a**) BCF-experiment and (**b**) NF-experiment. (**c,d**) are the same as (**a,b**) but for the covariance of 50 hPa westerly wind with the 50 hPa NAMI Only significant differences at 95%-level according to a covariance test are shaded.

Finally, in Supplementary Figure S1b, ‘BSF-NF’ should read ‘BCF-NF’. The correct Supplementary Figure S1 appears below as Figure 3.

**Figure 3:**
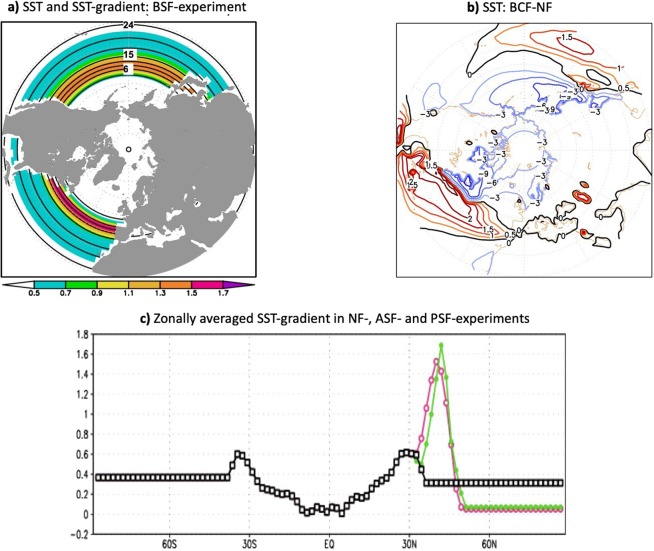
Lower-boundary forcing in the zonally symmetric SST-front experiments. The January SST-forcing and corresponding meridional SST-gradient shown for (**a**) both (Atlantic and Pacific) Symmetric Fronts (BSF)-experiment. (**b**) represents the SST differences between the configuration with realistic Atlantic and Pacific SST-fronts (BCF) and non-front (NF) configuration. (**c**) represents the global zonally averaged SST-gradient used in NF-experiments (in black), experiments with zonally-symmetric Pacific SST-front (in pink) and experiments with zonally-symmetric Atlantic SST-front (in green).

