# Supplementary material for: Key Role of the Ocean Western Boundary currents in shaping the Northern Hemisphere climate
Source: Sci Rep. 2019 Feb 28;9:3014. doi: 10.1038/s41598-019-39392-y (PMC6395655; doi:10.1038/s41598-019-39392-y)
Supplement: Supplementary file 1 — Key Role of the Ocean Western Boundary currents in shaping the Northern Hemisphere climate. [file 41598_2019_39392_MOESM1_ESM.docx]

Key Role of the Ocean Western Boundary currents in shaping the Northern Hemisphere climate.

* Nour-Eddine Omrani^1^, Fumiaki Ogawa^1^, Hisashi Nakamura^2^, Noel Keenlyside^1,3^, Sandro W. Lubis^4^, and Katja Matthes^5, 6^

^1^Geophysical Institute, University of Bergen, and Bjerknes Center for Climate Research, Bergen, Norway

^2^Research Center for Advanced Science and Technology, University of Tokyo, Tokyo, Japan, and Japan Agency for Marine-Earth Science and Technology, Yokohama, Japan

^3^Nansen Environmental and Remote Sensing Center, Bjerknes Centre for Climate Research, Bergen, Norway

^4^Department of Geophysical Sciences, University of Chicago, Chicago, Illinois, USA

^5^Research Division Ocean Circulation and Climate, GEOMAR Helmholtz Centre for Ocean Research, Kiel, Germany.

^6^Christian-Albrechts-University of Kiel, Germany.

***Supplementary Figures:***

***
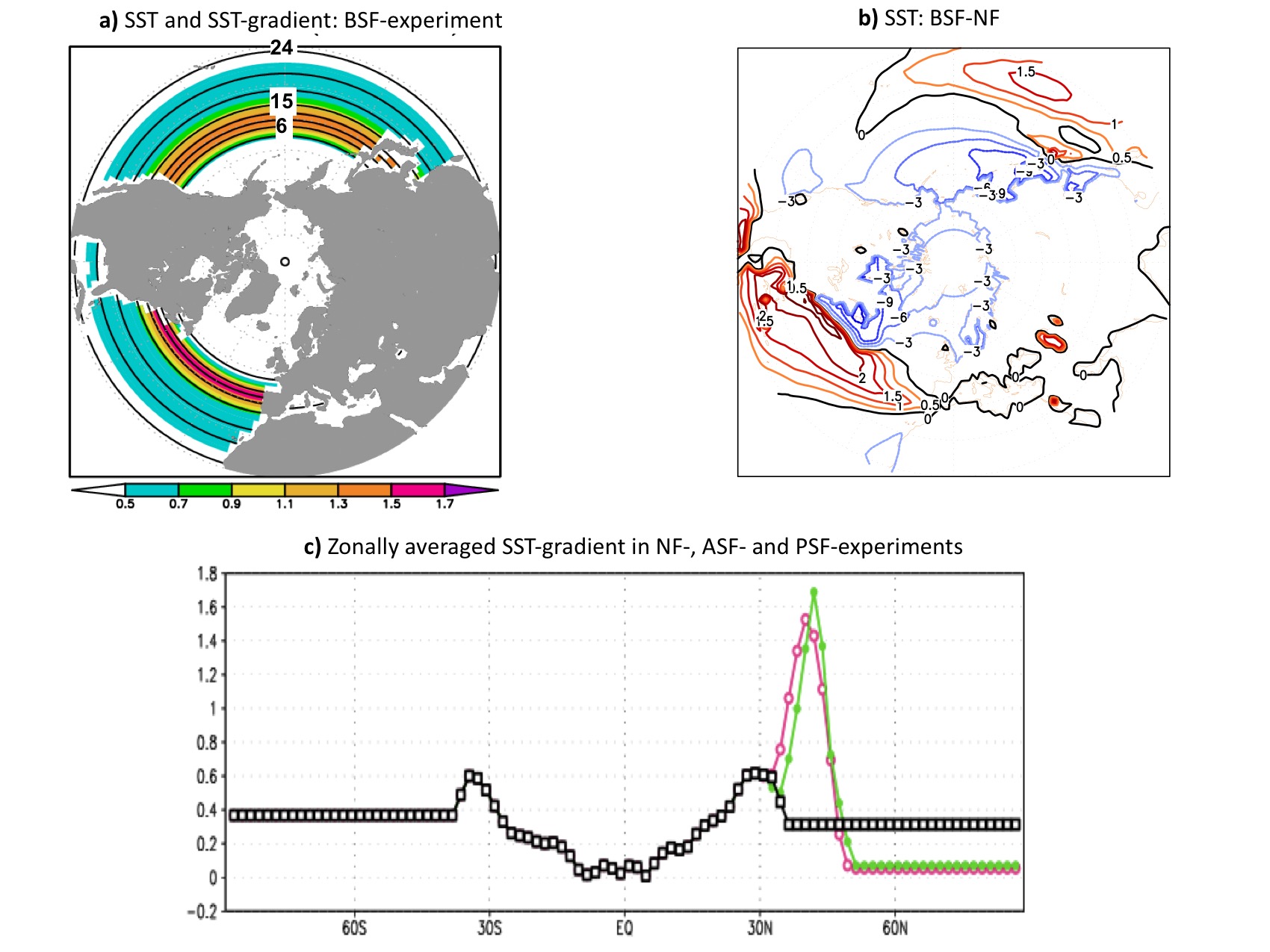
***

**SFig. 1. Lower-boundary forcing in the zonally symmetric SST-front experiments.** The January SST-forcing and corresponding meridional SST-gradient shown for (a) both (Atlantic and Pacific) Symmetric Fronts (BSF)-experiment. (b) represents the SST differences between the configuration with Atlantic and Pacific SST-fronts (BCF) and non-front (NF) configuration. (c) represents the global zonally averaged SST-gradient used in NF-experiments (in black), experiments with zonally-symmetric Pacific SST-front (in pink) and experiments with zonally-symmetric Atlantic SST-front (in green).


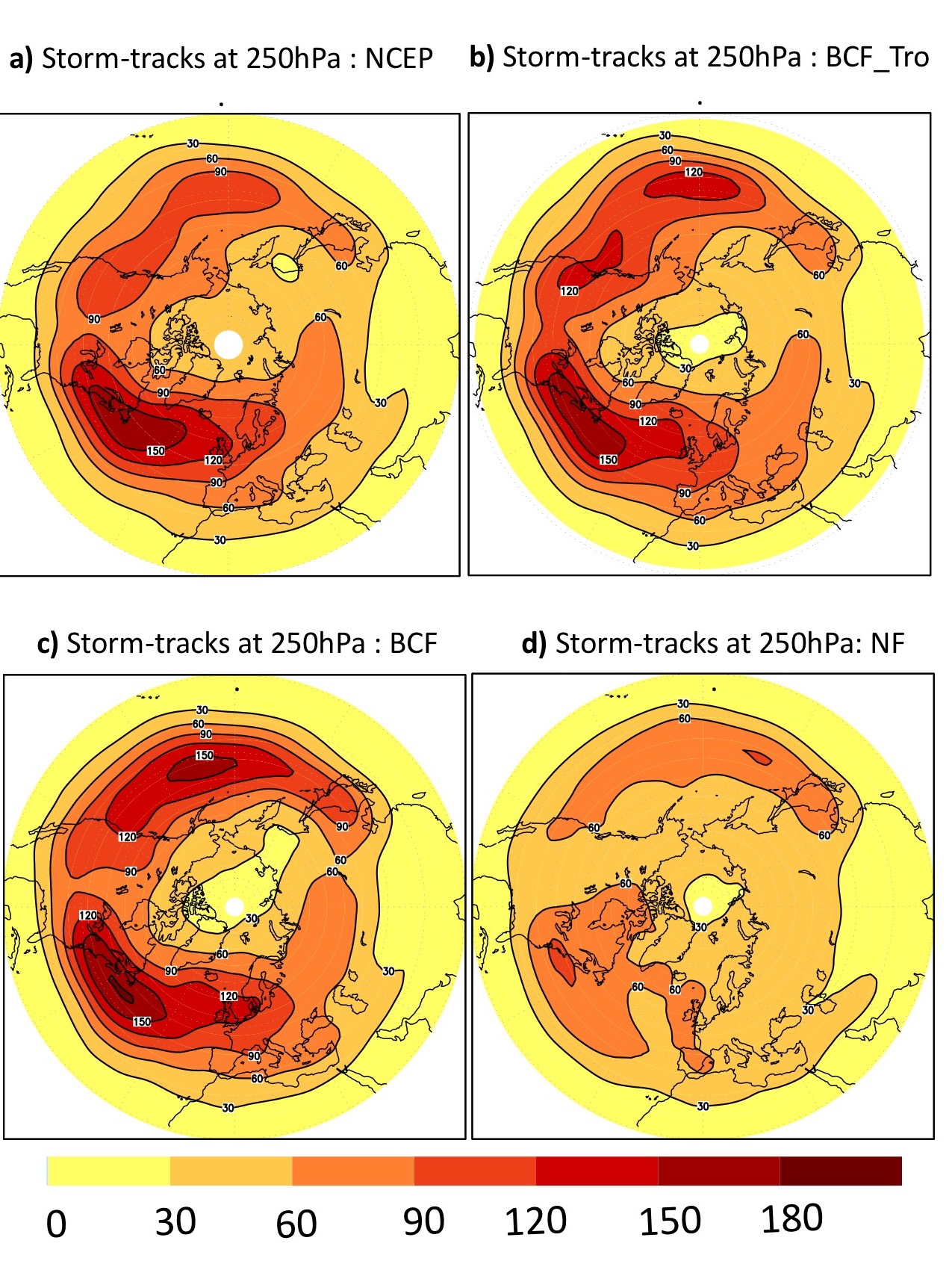


**SFig. 2. Winter climatology of the Storm-tracks:** (a), (b), (c) and (d) represent the wintertime storm-tracks at 250hPa for (a) the NCEP-reanalysis, (b) the BCF_Tro-experiment, (c) the BCF-experiment and (d) the NF-experiment.


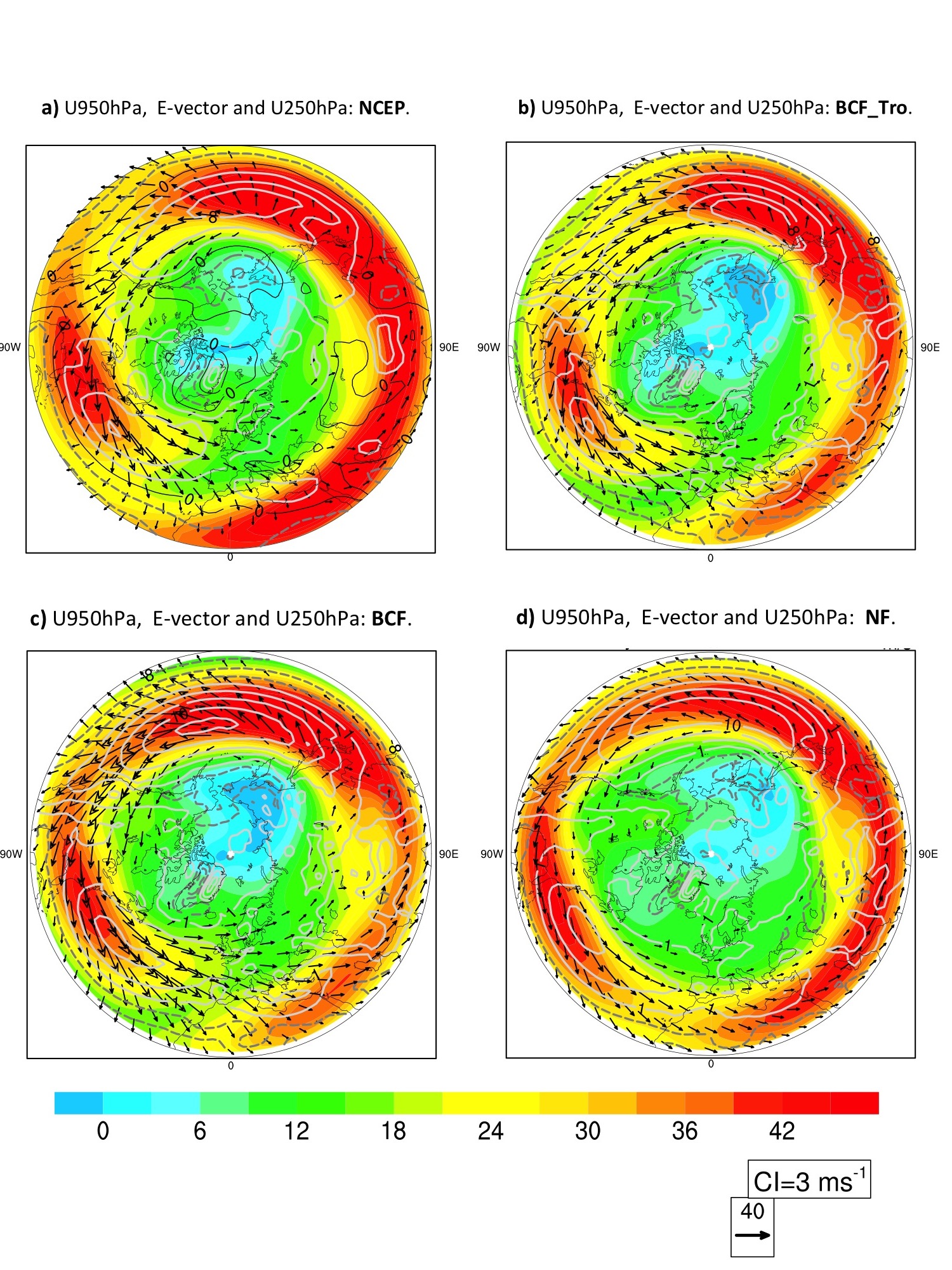


**SFig. 3. Winter Climatology of the wind and E-vector:** (a), (b), (c) and (d) represent the wintertime horizontal components of E-vector (vectors, see Method) superposed to the tropospheric zonal wind at 250hPa-level (shaded in m/s) and 950hPa (white contour, in m/s) for (a) the NCEP-reanalysis, (b) the BCF_Tro-experiment, (c) the BCF-experiment and (d) the NF-experiment.


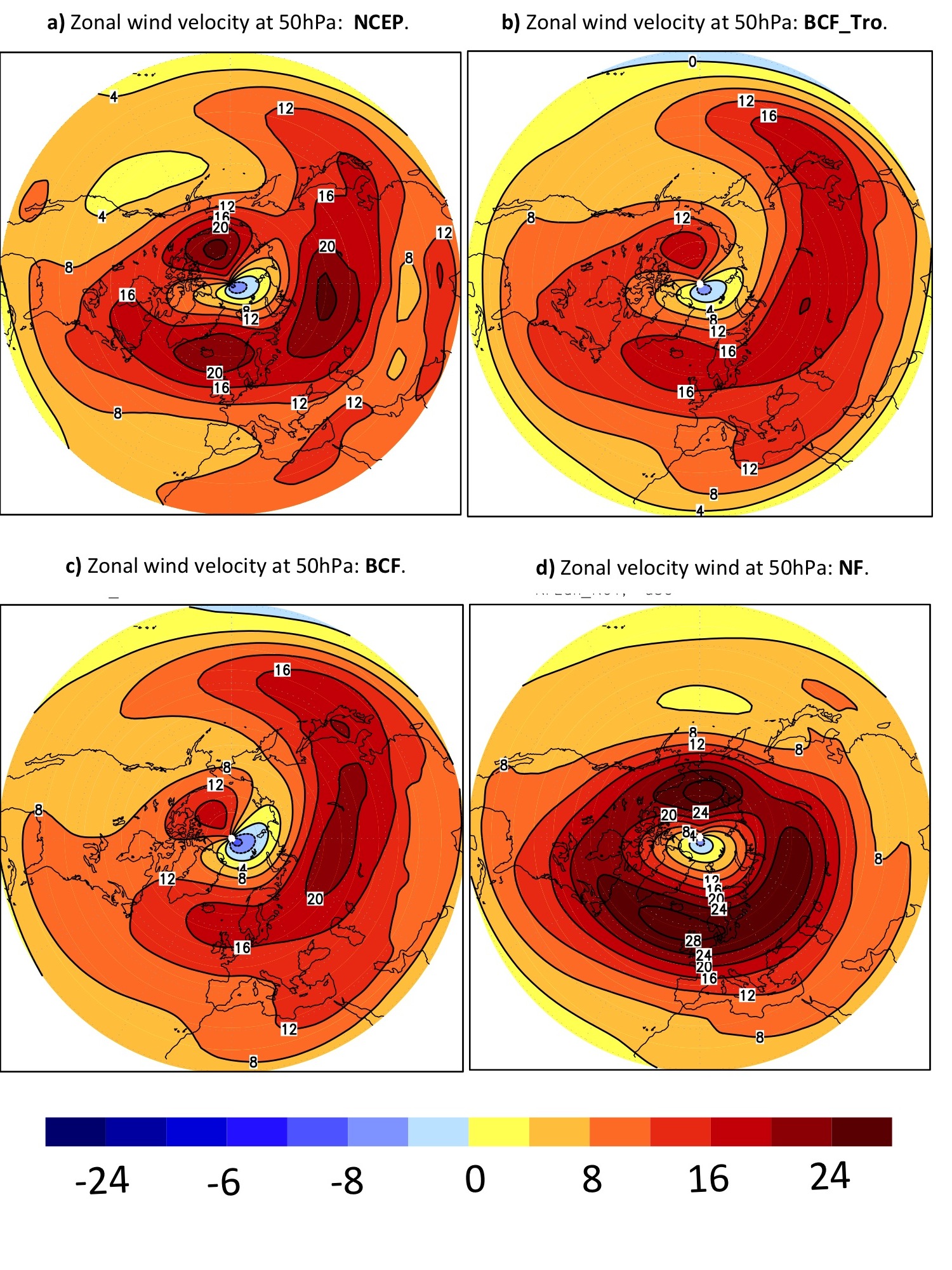


**SFig. 4. Winter climatology of stratospheric wind at 50hPa:** (a), (b), (c) and (d) represent the wintertime westerly wind at 50hPa (in m/s) for (a) the NCEP-reanalysis, (b) the BCF_Tro-experiment, (c) the BCF-experiment and (d) the NF-experiment

**
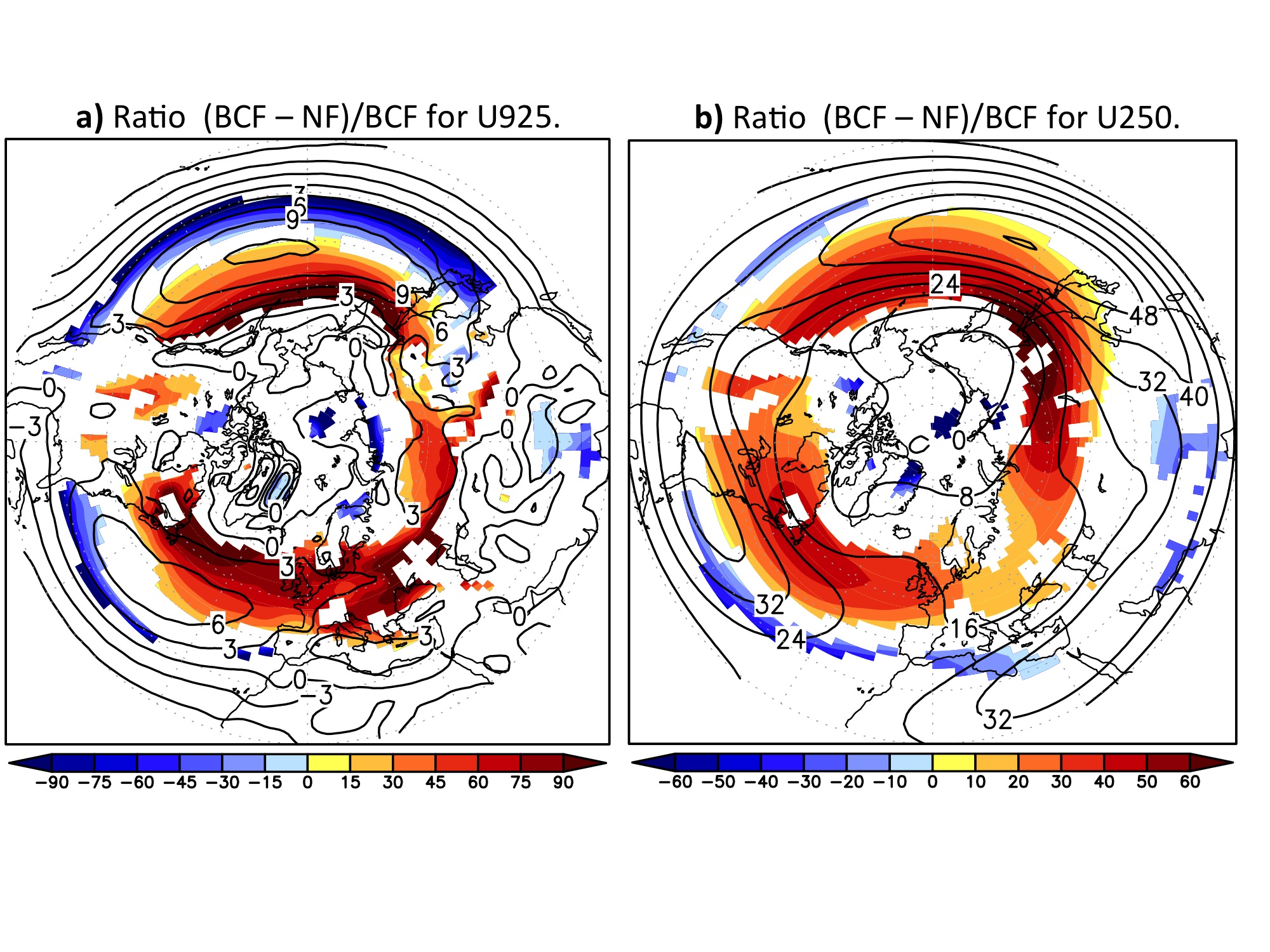
**

**SFig. 5:** **Comparison of the westerly wind response to its winter climatology.** (a) and (b) gives the wintertime (JFM) ratio (BCF – NF)/BCF of the zonal wind (in % of BCF-values, shading) in comparison with BCF (contour) at levels (a) 925hPa, and (b) 250hPa. The ratio (BCF – NF)/BCF is presented only in the region where the climatological wind in BCF is westerly.


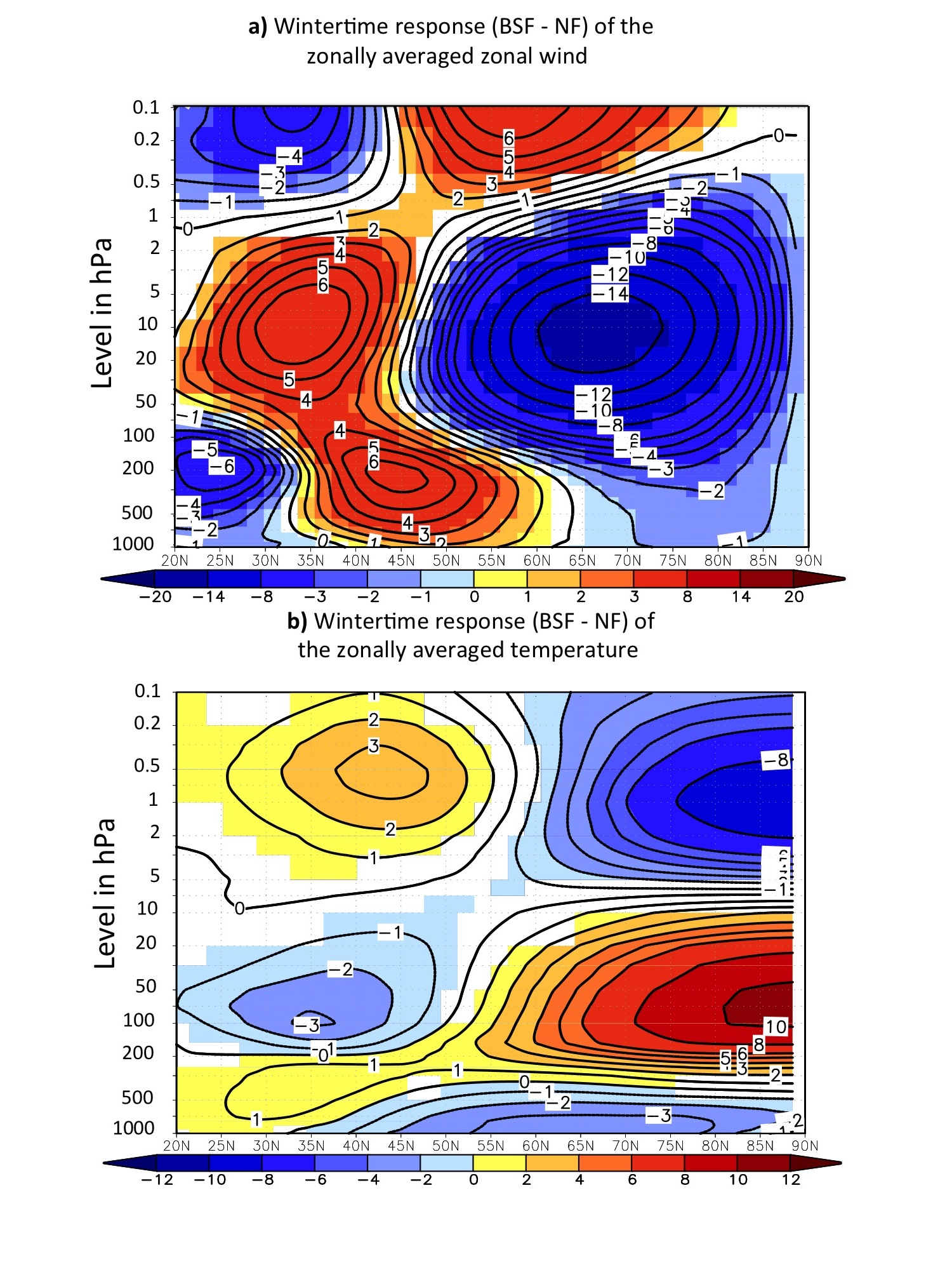


**SFig. 6:** **Impact of NH realistic SST-fronts on the zonally-averaged zonal wind and temperature.** The wintertime (JFM) difference between BCF- and NF-experiment of the zonally averaged zonal wind (a) and temperature (b). Only significant differences at 95%-level are shaded according to two-sided t-test.

**
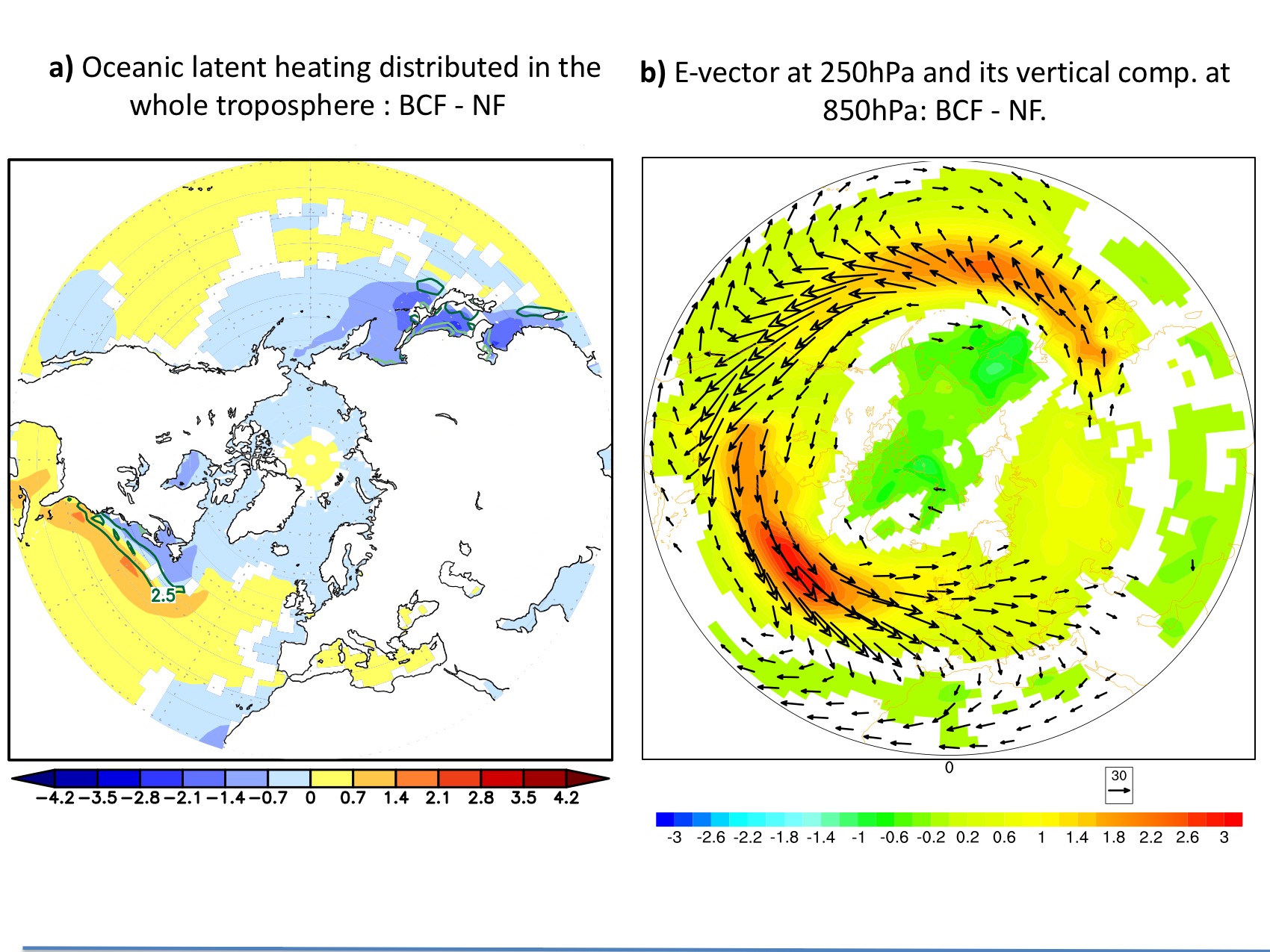
**

**SFig. 7: Latent heat flux impact and upward wave activity flux:**

(a) The difference BCF – NF in the wintertime (JFM) temperature tendency due to oceanic latent heat (LH, in °K/day), which is assumed to be distributed over the whole troposphere (1000 to 150hPa), superimposed on its reversed meridional gradient (contour in (K/day)/°latitude). Compared to the sensible heat fluxes, which maintain the baroclinicity in the lower troposphere, the LH maintains the baroclinicity in the whole troposphere, where it is released as condensation heat. The reversed gradients of temperature-tendencies in (a) are contoured in dark green for positive values and light green for negative values. (b) as (a) but for the upward flux of wave activity (shaded) superimposed on the E-vectors. Only significant differences at 95%-level are shaded according to two-sided t-tests.


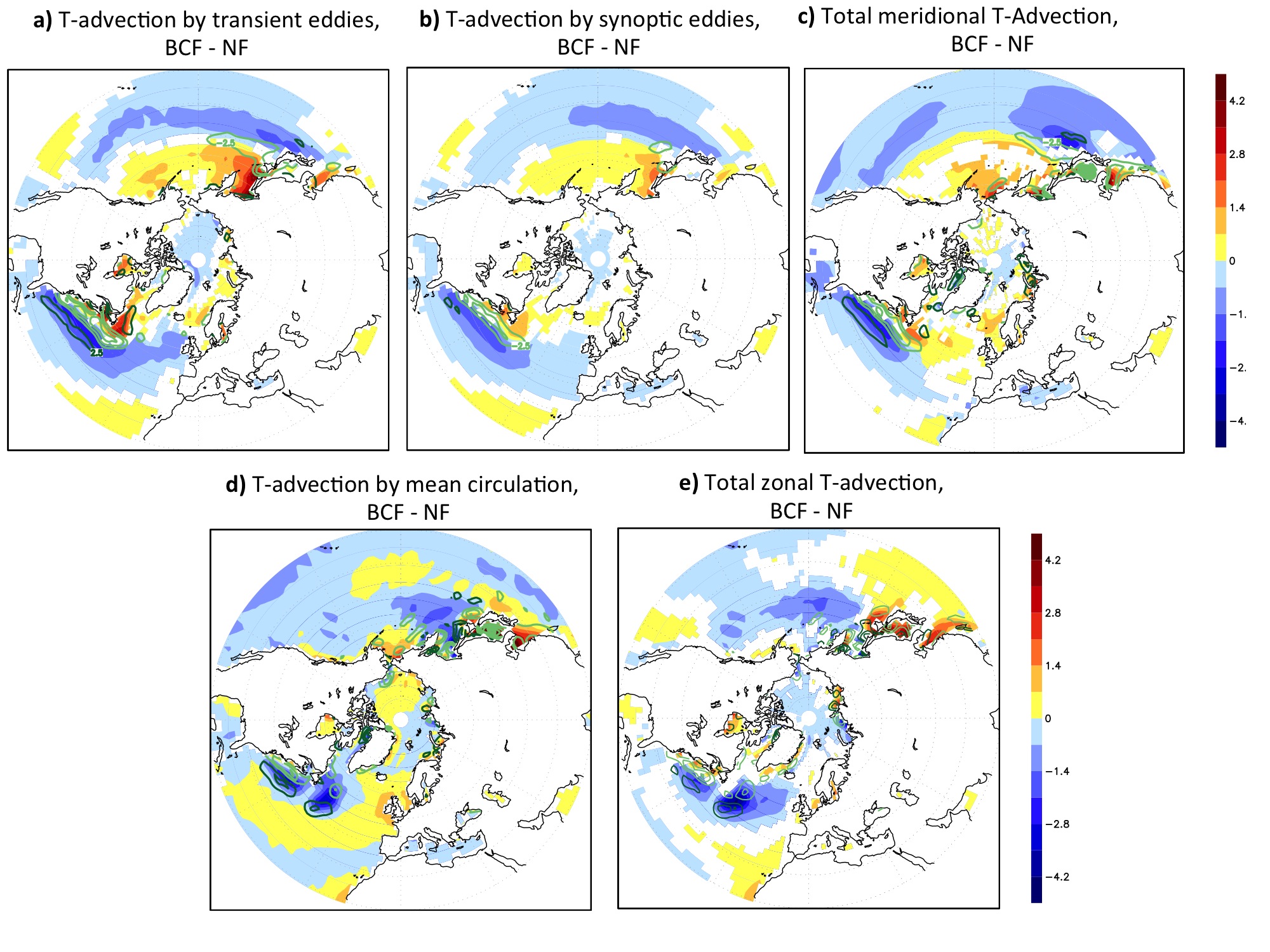


**SFig. 8: Impact of transient, baroclinic wave and zonal circulation on the response of T-advection.** (a), (b) and (c) compare the differences (BCF - NF) of the horizontal T-advection by transient eddies (defined as deviation from the time mean) in (a) with the advection by synoptic eddies (1-12day) in (b) and by total meridional T-advection in (c). (d) and (e) compare the T-advection of the mean circulation in (c) with the total zonal T-advection in (e). Only significant differences at 95%-level are shaded according to the two-sided t-test. Note that the horizontal transient T-advection can be explained mainly by the advection by the synoptic eddies, which explains most of the meridional T-advection. The horizontal T-advection by the mean circulation can be explained mainly the zonal T-advection. Since the zonal wind response to the SST-front is determined by synoptic-eddies feedback, the horizontal T-advection by the mean circulation can be regarded as an indirect impact of the baroclinic eddies.


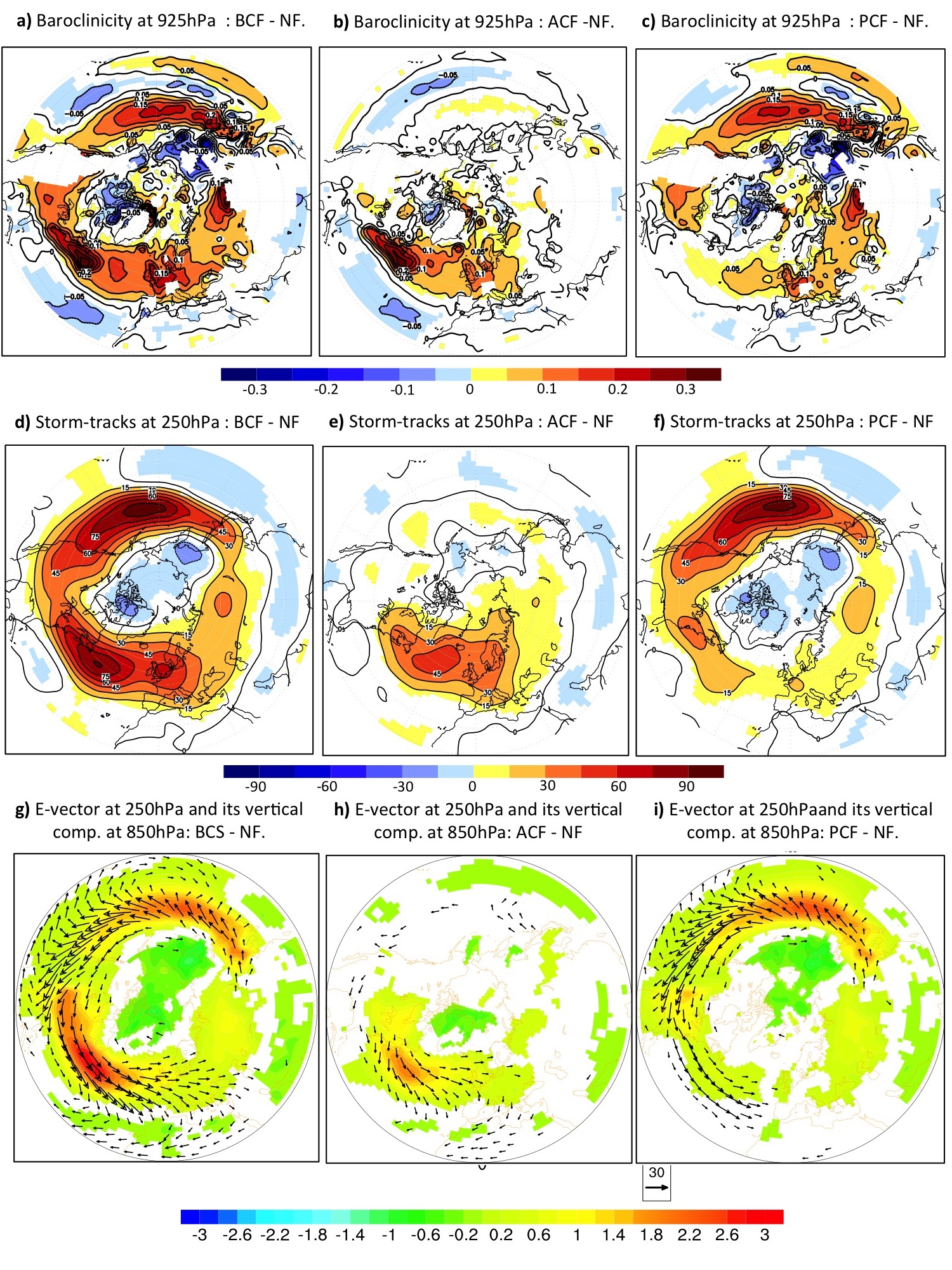


**SFig. 9: Contribution of the Atlantic and Pacific basins to the overall dynamical response to the realistic SST-fronts.** The wintertime (JFM) difference to NF-experiment of the lower-tropospheric baroclinicity (at 925hPa) presented for (a) BCF-experiment, (b) ACF-experiment and (c) PCF-experiment. (d), (e) and (f) are similar to (a), (b) and (c), but for the storm-tracks. (g), (h) and (i) are similar to (a), (b) and (c), but for the upward flux of wave activity (shaded) superimposed on the E-vectors. In all figures only significant differences at 95%-level are shaded according to the two-sided t-test. The baroclinicity values at level lower that the earth surface (e.g. mountains) are masked out.

**
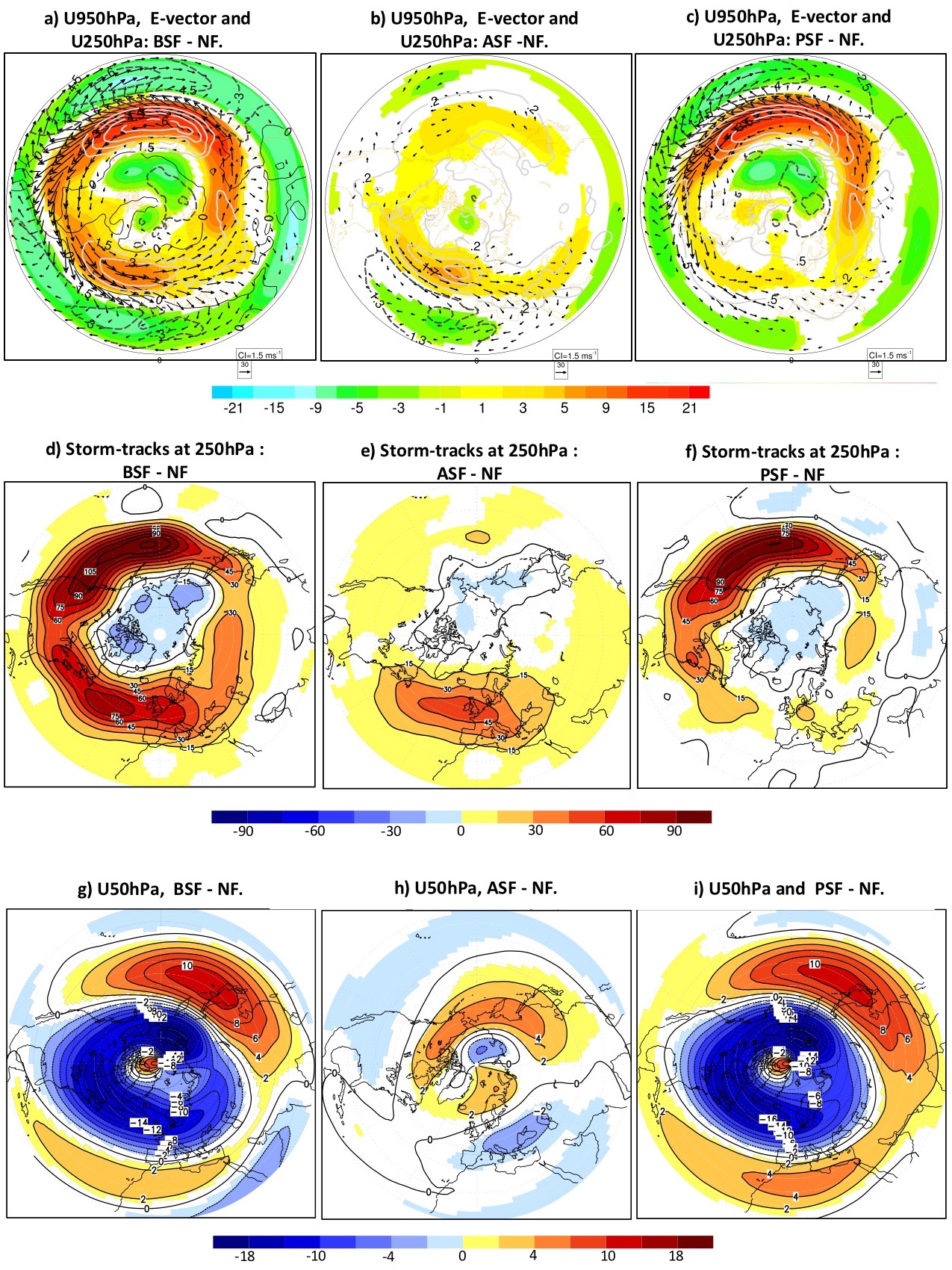
SFig. 10: Response to symmetric SST-front:**

(a), (b) and (c) are like Fig. 3c but for the response of the symmetric SST-front for (a) both Atlantic and Pacific SST-fronts (BSF-NF), (b) only Atlantic SST-Front (ASF-NF), and (c) only Pacific SST-front (PSF-NF). (d), (e) and (f) are similar to (a), (b) and (c) respectively but for the baroclinicity. (d), (e) and (f) are similar to (a), (b) and (c) respectively but for the stratospheric zonal wind at 50hPa.

The comparison between zonally symmetric SST-front experiments and the zonally asymmetric climatological SST-front experiments by comparing:

(1) SFig. 10a), 10b) and 10c) with Fig. 3c), Fig. 5a) and 5b)

(2) SFig. 10d), 10e) and 10f) with SFig. 9d), SFig. 9e) and SFig. 9f

and (3) SFig. 10g), 10h) and 10i) with Fig. 2d), 5c) and 5d)

reveals that the zonal asymmetry plays a minor role in the atmospheric response to the NH SST-frons.


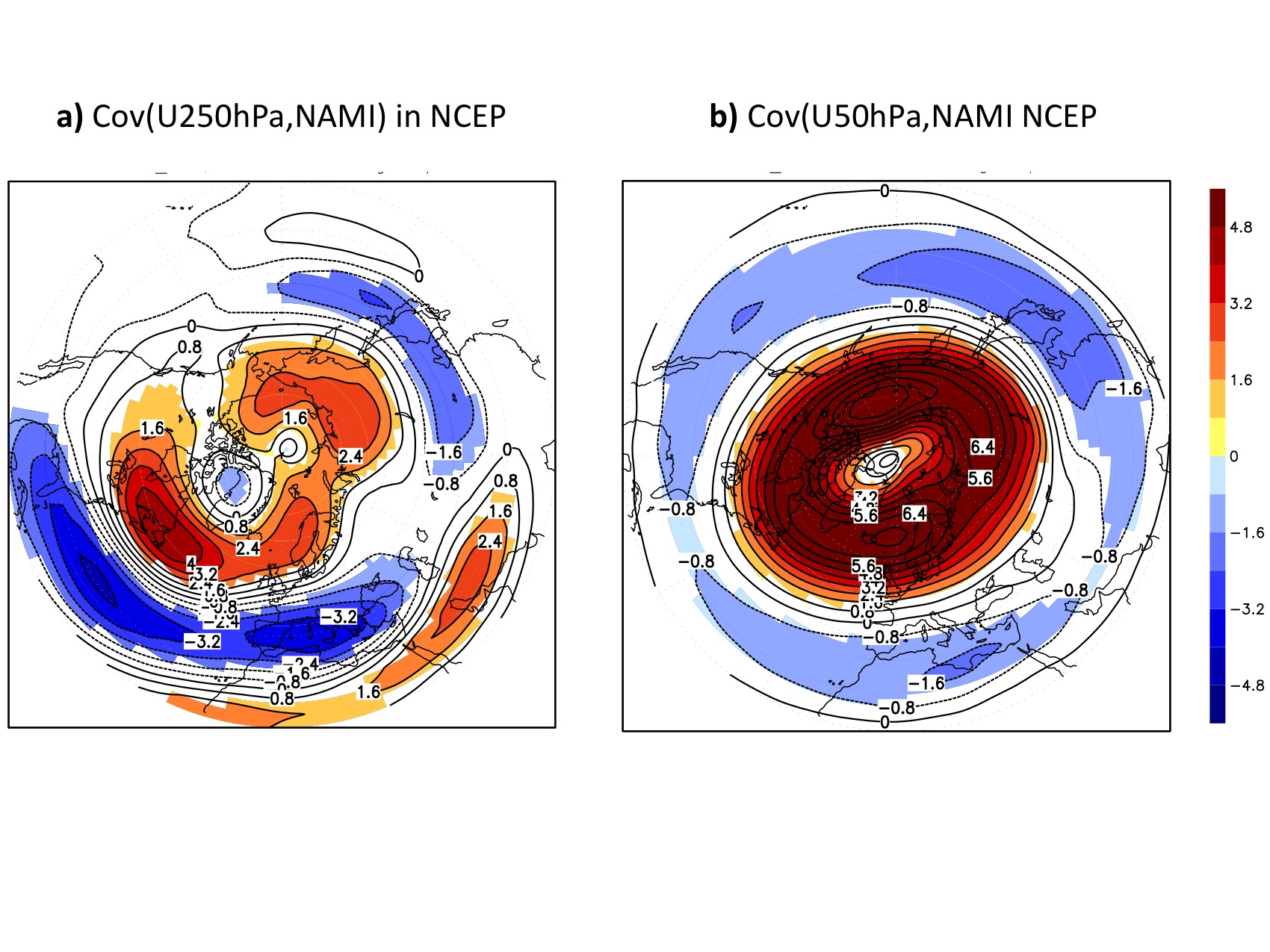


**SFig. 11: The stratospheric and tropospheric NAM in NCEP-reanalysis**: (a) is like Fig. 6a-b in the main text but for the NCE-reanalysis. (b) is like Fig. 6c-d but for the NCEP-reanalysis.
